# Dualistic insulator states in 1T-TaS_2_ crystals

**DOI:** 10.1038/s41467-024-47728-0

**Published:** 2024-04-23

**Authors:** Yihao Wang, Zhihao Li, Xuan Luo, Jingjing Gao, Yuyan Han, Jialiang Jiang, Jin Tang, Huanxin Ju, Tongrui Li, Run Lv, Shengtao Cui, Yingguo Yang, Yuping Sun, Junfa Zhu, Xingyu Gao, Wenjian Lu, Zhe Sun, Hai Xu, Yimin Xiong, Liang Cao

**Affiliations:** 1 Anhui Key Laboratory of Low-Energy Quantum Materials and Devices, High Magnetic Field Laboratory, HFIPS, Chinese Academy of Sciences, Hefei, 230031 P. R. China; 2 Changchun Institute of Optics, Fine Mechanics and Physics, Chinese Academy of Sciences, Changchun, Jilin 130033 P. R. China; 3 Key Laboratory of Materials Physics, Institute of Solid State Physics, HFIPS, Chinese Academy of Sciences, Hefei, 230031 P. R. China; 4 Department of Physics, School of Physics and Optoelectronics Engineering, Anhui University, Hefei, 230601 P. R. China; 5 PHI Analytical Laboratory, ULVAC-PHI Instruments Co., Ltd., Nanjing, 211110 Jiangsu P. R. China; 6 National Synchrotron Radiation Laboratory, University of Science and Technology of China, Hefei, 230026 P. R. China; 7 Science Island Branch of Graduate School, University of Science and Technology of China, Hefei, 230026 P. R. China; 8 State Key Laboratory of Photovoltaic Science and Technology, School of Microelectronics, Fudan University, Shanghai, 200433 P. R. China; 9 Collaborative Innovation Center of Advanced Microstructures, Nanjing University, Nanjing, 210093 P. R. China; 10 Shanghai Synchrotron Radiation Facility (SSRF), Zhangjiang Laboratory, Shanghai Advanced Research Institute, Chinese Academy of Sciences, 239 Zhangheng Road, Shanghai, 201204 P. R. China; 11 Hefei National Laboratory, Hefei, 230028 P. R. China; 12 Center of Materials Science and Optoelectronics Engineering, University of Chinese Academy of Sciences, Beijing, 100049 P. R. China

**Keywords:** Phase transitions and critical phenomena, Electronic properties and materials, Two-dimensional materials

## Abstract

While the monolayer sheet is well-established as a Mott-insulator with a finite energy gap, the insulating nature of bulk 1T-TaS_2_ crystals remains ambiguous due to their varying dimensionalities and alterable interlayer coupling. In this study, we present a unique approach to unlock the intertwined two-dimensional Mott-insulator and three-dimensional band-insulator states in bulk 1T-TaS_2_ crystals by structuring a laddering stack along the out-of-plane direction. Through modulating the interlayer coupling, the insulating nature can be switched between band-insulator and Mott-insulator mechanisms. Our findings demonstrate the duality of insulating nature in 1T-TaS_2_ crystals. By manipulating the translational degree of freedom in layered crystals, our discovery presents a promising strategy for exploring fascinating physics, independent of their dimensionality, thereby offering a “three-dimensional” control for the era of slidetronics.

## Abstract (web summary)

While monolayer of 1T-TaS_2_ is considered to be a Mott insulator, the nature of the bulk insulating state is debated. Here the authors introduce a ladder-type structures with fractional misalignment of adjacent layers, showing that it becomes a Mott insulator due to decoupling between the layers.

## Introduction

**Fig. 1:**
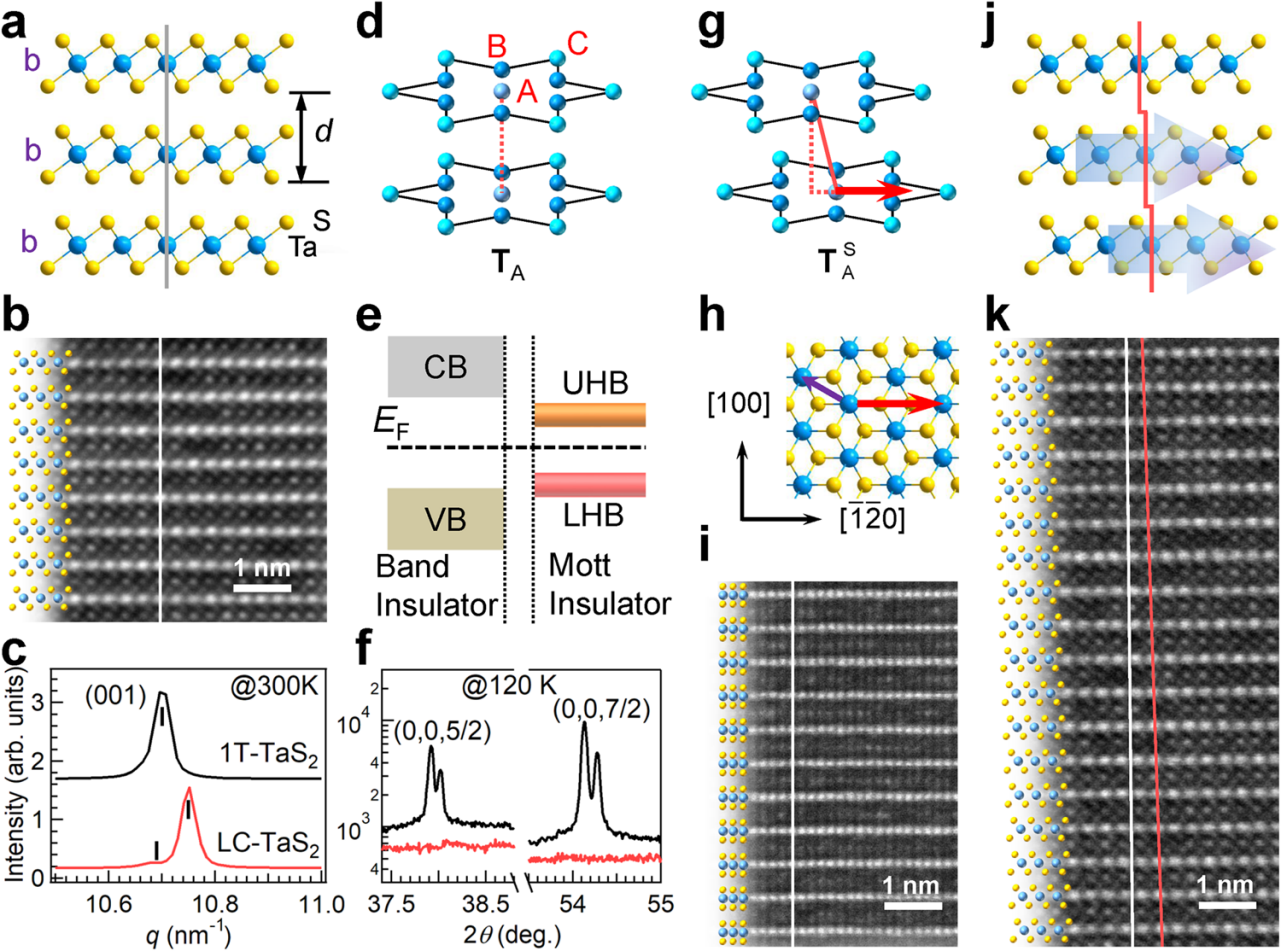
Variation in the atomic structure and insulating nature. **a** Schematic side view of 1T-TaS_2_ crystals and (**b**) corresponding [100] cross-sectional HAADF-STEM image. **c** Synchrotron-based XRD spectra around the (001) diffraction peak collected at room temperature. **f** The out-of-plane superlattice and reflections for a 1T-TaS_2_ crystal collected at 120 K. The peak splitting is attributed to the Cu K_α1_ and K_α2_ X-ray split. Illustration of (**d**) the David-stars T_A_ stacking and (**g**) the T^S^_A_ stacking associated with (**j**) the laddering stack structure in LC-TaS_2_ crystals verified by HAADF-STEM images collected along (**k**) [100] and (**i**) [$\bar{12}$0] directions. The white vertical lines in panels (**b,** **k**, **i**) indicate the *c*-direction. The red solid line in panel (**k**) highlights the atomic misalignment. **h** Schematic top view of monolayer octahedral TaS_2_. The red and purple arrows indicate the [$\bar{12}$0] and [110] directions, respectively. **e** Schematic energy band diagrams depicting a band-insulator and a Mott-insulator. CB, VB, UHB, and LHB denote the conduction band, valence band, upper Hubbard band, and lower Hubbard band, respectively.

The discovery of Mott-insulating states holds significant relevance for unconventional superconductors and quantum spin liquid phase (QSL), showing promise for applications in quantum computing^1–4^. The Mott-insulating state in the layered octahedral 1T-TaS_2_ crystals, first proposed four decades ago^5^, has led to extensive investigations into the intertwined correlations among Mott-insulator, charge density wave (CDW), and superconducting states^6–9^. The Mott-insulating nature is widely accepted in isolated monolayer 1T-TaS_2_ due to the preserved in-plane David-stars in the commensurate CDW (C-CDW) state and the absence of interlayer coupling^10–13^. However, in the past decade, a plot twist has emerged in understanding the insulating behavior in bulk 1T-TaS_2_ crystals. Theoretical and experimental studies have introduced the concept of the band-insulator mechanism, considering the key role of the David-stars dimerization associated with specific on-top David-stars T_A_ stacking configurations (Fig. 1d)^10,13–18^. However, the theoretically predicted out-of-plane metallic state has not been observed in transport experiment^10,14–16,19^. And discrepancies exist between microscopic and macroscopic properties^17,20–22^. For instance, the recent findings of the band-to-Mott insulating phase transition at a narrow temperature window in 1T-TaS_2_ crystals upon heating have not fully manifested in the macroscopic transport measurements yet^22,23^. Along with anomalies like the absence of long-range magnetic order and unexpected emergence of superconducting states^6,7,18,24–27^, these complexities complicate the understanding of the physics of this system, making it challenging to reach a definitive conclusion about the insulating nature of 1T-TaS_2_ crystals.

As the surface-to-volume ratio increases in layered materials, surface and interface effects gain greater significance. The recent discovery of sliding ferroelectricity in two-dimensional (2D) layered homostructures, achieved by controlling the translational degrees of freedom^28–32^, naturally prompts a fundamental question: Can stacking modulation be extended to the three-dimensional (3D) counterpart? And how does lattice translation between adjacent layers in 3D layered crystals impact their electronic structures?

In this study, we introduced a laddering stack structure (Fig. 1j) in 1T-TaS_2_ crystals by deliberately creating fractional misalignment of adjacent layers, leading to a misaligned David-stars T^S^_A_ stacking (Fig. 1g). This unique approach unveiled a distinct insulating state, shedding light on switching between Mott-states and band-insulating states through interlayer stacking and coupling manipulation. With interlayer coupling, the dominance shifts towards the 3D band-insulating states, whereas interlayer decoupling, achieved through the structuring of laddering interlayer sliding, favors the prevalence of 2D Mott-insulating states. This discovery provides compelling evidence of the dualistic insulating nature of 1T-TaS_2_ crystals. Furthermore, extending this laddering stack structure to other layered materials with similar structural characteristics of intralayer stiffness and interlayer slipperiness opens up exciting possibilities to explore fascinating physics and intriguing properties regardless of their dimensionality.

## Results

### Laddering interlayer sliding and structural characterization

The laddering stack structure is verified using aberration-corrected high-angle annular dark-field scanning transmission electron microscopy (HAADF-STEM), as shown in Fig. 1k. This unique structure features a routine sandwiched unit, whereas a periodically misaligned interlayer stacking arrangement along the out-of-plane *c*-direction (Fig. 1j) distinguishes it from the well-established vertical alignment of Ta-atoms between adjacent layers in 1T-TaS_2_ (bb-stacking in Fig. 1a, b and Supplementary Fig. 1)^33^. The interlayer sliding occurs along the [$\bar{12}$0] direction within the *ab*-plane guided by red arrows in Fig. 1h, g, as determined from the corresponding HAADF-STEM image (Fig. 1i). Viewing along the [100] direction, a 20-layer periodicity is discerned from a large-scale HAADF-STEM image (see Supplementary Fig. 2). Considering the [$\bar{12}$0] direction interlayer sliding, the Ta-atoms in the upper layer are expected to align with the Ta-atoms in the lower layer after a 40-layer periodicity, corresponding to a translational shift of ~0.015 nm per layer. The 40-layer periodicity is confirmed by the HAADF-STEM image along [110] direction (Supplementary Fig. 3). Here, we name the crystals with octahedral coordinated TaS_2_ units stacking in a laddering configuration as LC-TaS_2_.

The interlayer spacing *d* is determined by synchrotron-based X-ray diffraction (XRD) spectroscopy at room temperature (Fig. 1c). The scattering vector *q* for LC-TaS_2_ is determined to be 10.75 nm^−1^, corresponding to a *d* = 0.584 nm (calculated using *d* = 2π/*q*). This *d* value is slightly smaller than 0.587 nm (*q* = 10.70 nm^−1^) for 1T-TaS_2_^34^, indicating a slight out-of-plane contraction. A shoulder with a lower *q* = 10.69 nm^−1^ originates from residual bb-stacking configurations. It is not surprising that a complete transition to the metastable laddering stack structural phase remains challenging, given that local fluctuation favors the relatively more stable 1T-phase^35,36^.

To understand the influence of laddering stack structure on interlayer dimerization, the XRD measurements were performed using Cu K_α_. Figure 1f displays the superlattice reflections collected at 120 K, and the (00 *l*) indexed diffraction peaks are presented in Supplementary Fig. 4. In the 1T-TaS_2_ crystal, interlayer dimerization associated with David-stars ordered array results in half-integer (0,0,5/2) and (0,0,7/2) reflections at ~37.9° and ~54.1°, consistent with previous findings^22^. The absence of these superlattice reflections in the LC-TaS_2_ crystal implies the collapse of interlayer dimerization, either caused by the collapse of CDW states or the absence of a David-stars ordered array.

### Electron localization at the David-star center

**Fig. 2:**
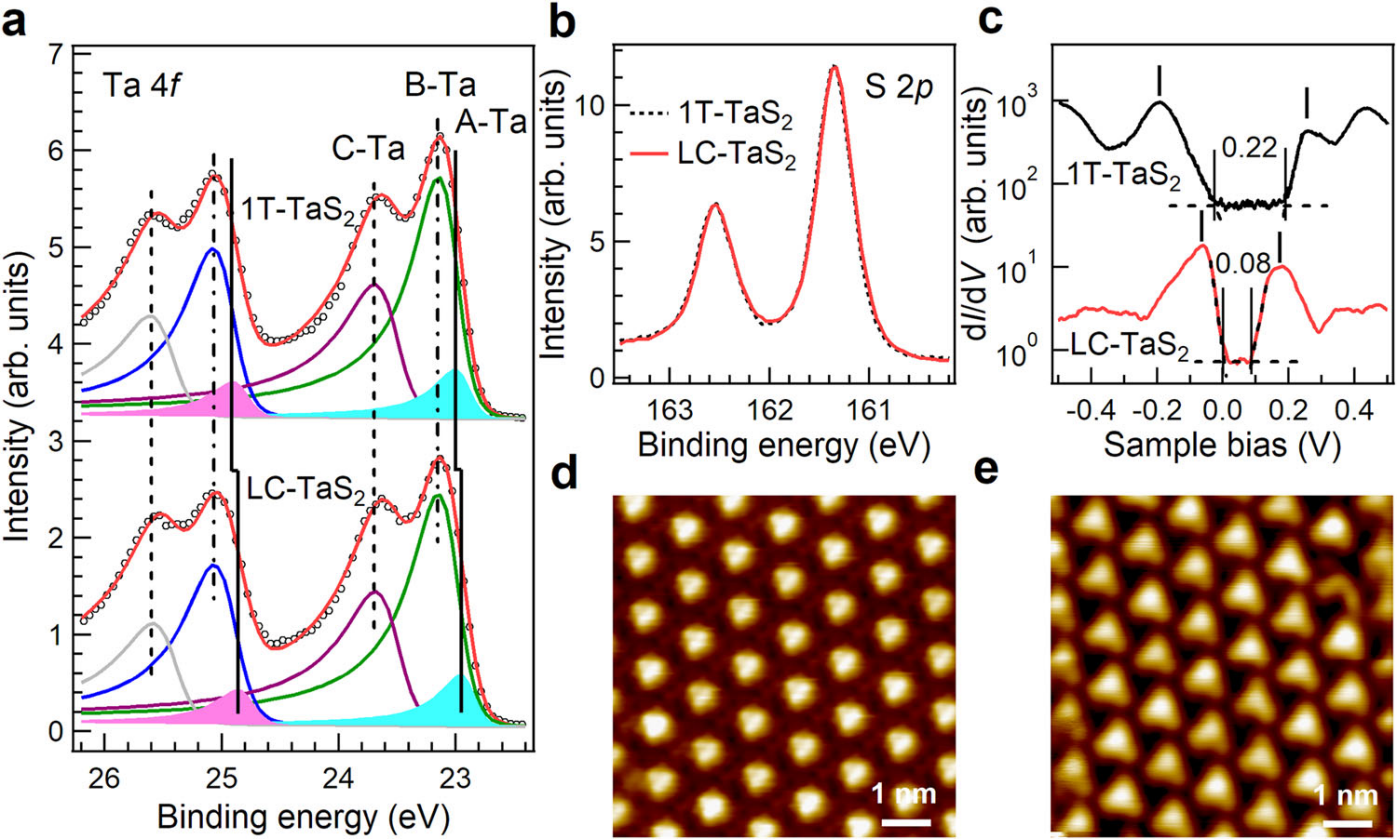
Evolution of chemical composition and electronic structures. **a** Ta 4*f* with corresponding fitting curves and (**b**) S 2*p* core-level spectra measured at room temperature using a photon energy of 240 eV. **c** Spatially averaged d*I*/d*V* spectra with logarithmic intensity scale conducted at David-star centers for 1T-TaS_2_ and LC-TaS_2_ crystals at 4.5 K. The curves are vertically shifted for clarity with zero conductance marked by horizontal dashed lines. STM images of the C-CDW phase for (**d**) 1T-TaS_2_ and (**e**) LC-TaS_2_ crystals conducted at 4.5 K.

To understand the influence of the laddering interlayer sliding on charge redistribution within the David-stars, synchrotron-based photoemission spectroscopy (PES) was conducted at room temperature, where 1T-TaS_2_ is in the nearly-commensurate CDW (NC-CDW) phase. Within each David-star, there is in-plane electron transfer from outer C-/B-Ta-atoms to the central A-Ta-atoms (Fig. 1d)^37,38^. The distinct core-hole screening effect, resulting from unequal local electron density at specific Ta-sites, leads to further splitting of the Ta 4*f*_7/2_ peak (Fig. 2a). Three components at ~23.64, ~23.10, and ~22.97 eV with an intensity ratio of ~6:6:1 are extracted for 1T-TaS_2_. The binding energies (BEs) of C-Ta and B-Ta features for LC-TaS_2_ remain unchanged, indicating the persistence of the CDW states. However, a lower BE shift of the A-Ta feature (~0.05 eV), which is appearance seen in Supplementary Fig. 5, indicates enhanced electron density and localization at A-Ta-atoms. This effect is most likely caused by interlayer decoupling^15^, resulting from laddering interlayer sliding.

An alternative explanation for the lower BE feature, attributed to the 2*H*-TaS_2_ phase or Ta^3+^ species associated with S-vacancy defects^39^, can be ruled out based on the following evidences:

(i) The S 2*p* spectral profile for LC-TaS_2_ crystals remains almost unchanged (Fig. 2b), indicating the absence of 2*H*-TaS_2_. Otherwise, an additional feature at ~160.80 eV would be detected, as we reported previously^39^.

(ii) The comparable intensity ratio of Ta/S for both 1T-TaS_2_ (~0.91) and LC-TaS_2_ (~0.92) crystals indicates a constant chemical stoichiometry.

### Duality of insulating states

To further understand the influence of the laddering interlayer sliding on the properties of insulating states and CDW order presented at low temperatures, scanning tunneling microscopy/spectroscopy (STM/STS) measurements were conducted at 4.5 K. The STM images of both 1T-TaS_2_ (Fig. 2d) and LC-TaS_2_ (Fig. 2e) crystals reveal the well-resolved C-CDW phase accompanied by a commensurate √13 × √13 triangular David-star superlattice, where each bright spot corresponds to one David-star. Remarkably, the long-range CDW order remains intact at the surface of LC-TaS_2_ crystals. The persistence of David-stars, along with the absence of out-of-plane superlattice reflections (Fig. 1f), implies the absence of out-of-plane ordered array of Davis-stars.

Interestingly, the profiles of the corresponding d*I*/d*V* spectra change dramatically (Fig. 2c). For the as-grown 1T-TaS_2_ crystal, two distinct peaks centered at approximately −0.19 and 0.26 eV feature a peak-to-peak energy gap with Δ ~0.45 eV and an edge-to-edge energy gap of ~0.22 eV, consistent with previous experimental results^8,9,11,21,39,40^. However, for the LC-TaS_2_ crystal, two in-gap states appear at −0.06 eV and 0.18 eV, yielding a narrower energy gap of Δ ~0.24 eV and an edge-to-edge gap of ~0.08 eV. These pronounced differences in edge-to-edge gap features between 1T-TaS_2_ and LC-TaS_2_ crystals persist consistently across the surface, as shown in Supplementary Fig. 6d, f. The Δ~0.24 eV closely matches the theoretically predicted Mott-gap of ~0.2 eV for monolayer 1T-TaS_2_^10^, indicating that this insulating state is induced by Mott localization. Nevertheless, this value is smaller than the experimentally observed energy gap of ~0.45 eV in molecular beam epitaxy growth monolayer 1T-TaS_2_ on a graphene/SiC surface^41^, implying a larger screening of Coulomb repulsion in a 3D structure compared to a 2D monolayer. The STS spectra with a narrow energy gap have also been theoretically stimulated and experimentally observed on the cleaved 1T-TaS_2_ surface with specific David-stars T_C_ stacking^13,20,21,42^, suggesting their common origin of interlayer decoupling. Here, T_C_ refers to the vertical alignment between A-Ta-atoms and C-Ta-atoms of the adjacent David-stars. These findings imply that the two times larger gap of the as-grown 1T-TaS_2_ crystal can be attributed to a distinct mechanism, for instance, band-insulating.

### Flat band structure and DFT calculation

**Fig. 3:**
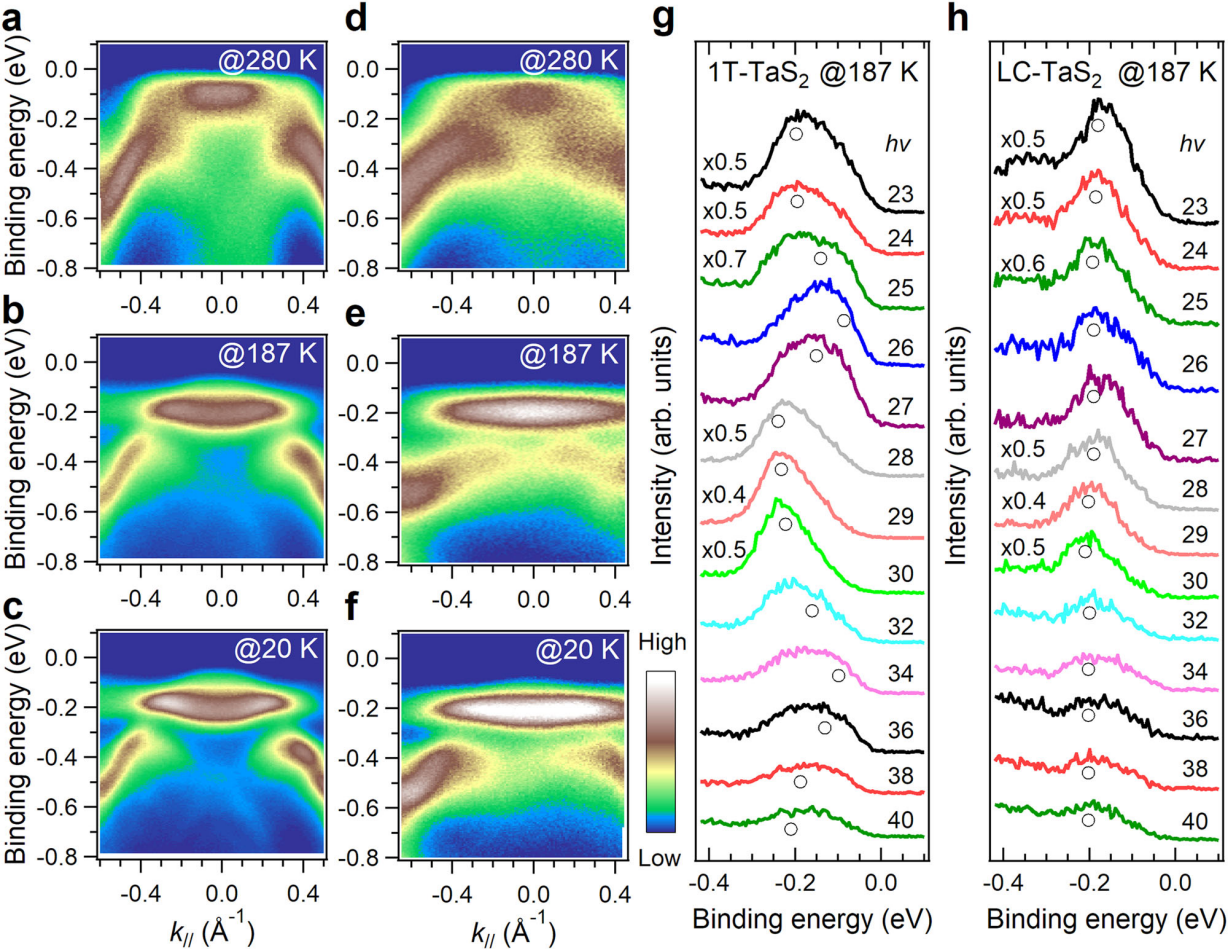
Band dispersions along the *k*_//_ and *k*_z_ direction in different electronic states. The ARPES data taken along the Γ-Μ/*k*_//_ direction (see Supplementary Fig. 7a, b) for (**a**–**c**) 1T-TaS_2_ and (**d**–**f**) LC-TaS_2_ at reducing temperatures of 280 K (NC-CDW phase), 187 K (C-CDW phase), and 20 K (insulating phase) with a photon energy of 22 eV. Photon energy dependence of energy distribution curves (EDCs) in the C-CDW phase collected at the Γ-point and 187 K for (**g**) 1T-TaS_2_ and (**h**) LC-TaS_2_. The opened circles represent the fitted band positions.

To gain insights into the nature of insulating states, the temperature-dependent band structures were investigated by using synchrotron-based angle-resolved PES (ARPES). The results for 1T-TaS_2_ crystals are consistent with previous findings^22,43^, providing a reliable reference for comparison. In the NC-CDW phase range at 280 K, both 1T-TaS_2_ and LC-TaS_2_ exhibit a flat band that emerges near the Fermi level (*E*_F_) (Fig. 3a, d). As the temperature decreases to 187 K, the flat bands shift away from *E*_F_ and center at ~−0.2 eV (Fig. 3b, e), resulting in an increase in the energy gap.

To investigate the band dispersion along the *k*_z_ direction, photon energy-dependent measurements were conducted at 187 K. In 1T-TaS_2_, the peak position shifts from −0.23 to −0.09 eV (Fig. 3g), whereas for LC-TaS_2_, the dominant peak at ~−0.2 eV exhibits less photon energy dependence (Fig. 3h). These characteristics were similar to those reported in 1T-TaS_2_ during a band-to-Mott insulating phase transition triggered by temperature^22^. In contrast, the observed characteristics in LC-TaS_2_ remain unchanged at 20 K (Fig. 3f and Supplementary Fig. 7d), indicating the insensitivity of the electronic states to temperatures and confirming their intrinsic nature. This feature, along with a distinct interlayer distance difference between LC- and 1T-TaS_2_ crystals, implies that the periodic laddering stack structure is unlikely to be the structural origin for the heating-triggered surface intermediate Mott-insulator phase^22,44^. However, the possibility of an interlayer atomic sliding confined to the surface region (5–8 layers), such as the irregular sliding observed in layered bulk PbI_2_^45^, cannot be ruled out.

**Fig. 4:**
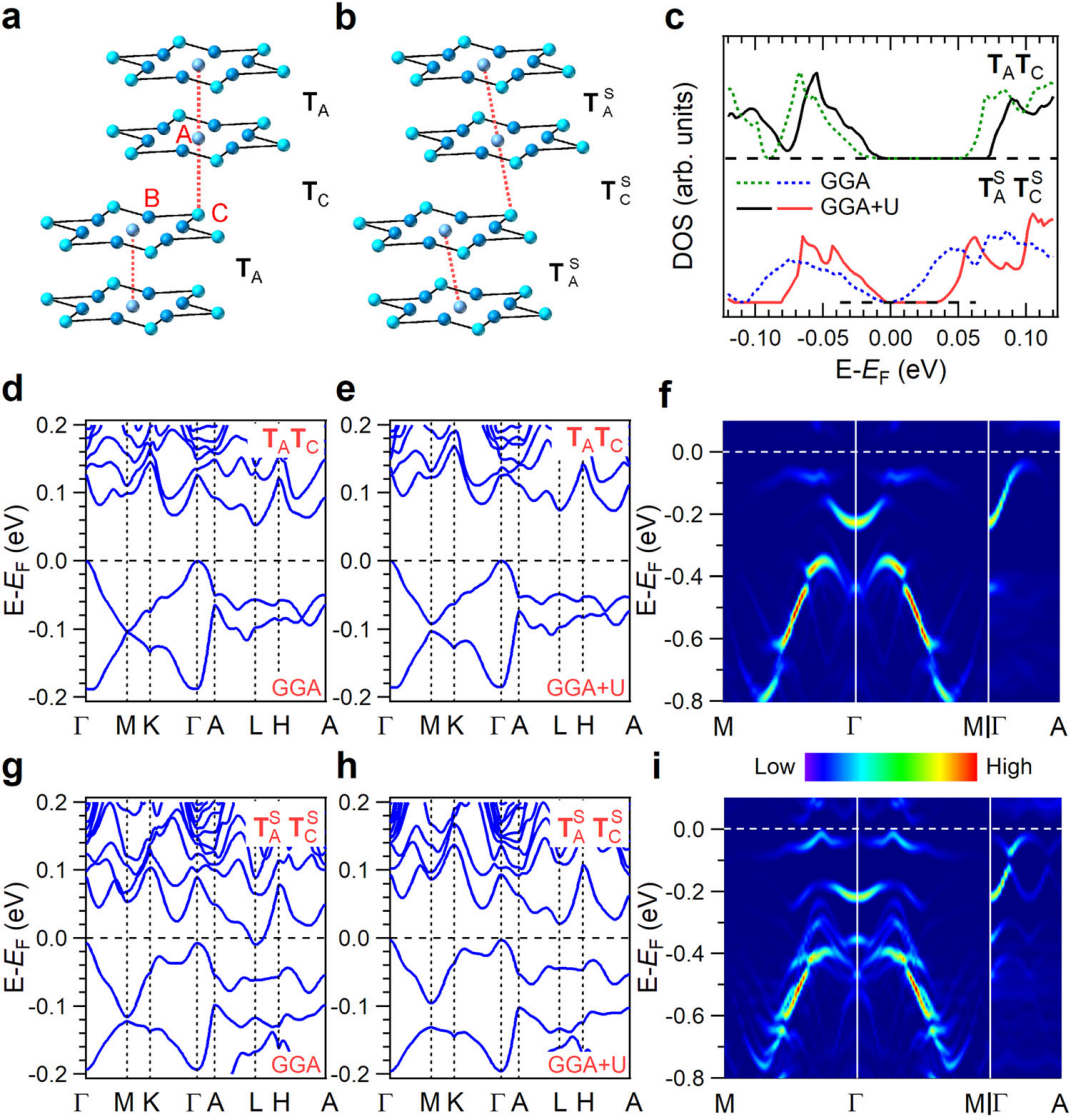
DFT calculated band structures. Schematic diagrams illustrating (**a**) the T_A_T_C_ and (**b**) T^S^_A_T^S^_C_ David-stars stacking sequence. **c** Density of States (DOS) for the T_A_T_C_ and T^S^_A_T^S^_C_ stacking. Band structures near the *E*_F_ for T_A_T_C_ stacking calculated using (**d**) generalized gradient approximation (GGA) and (**e**) GGA + U method. Band structure for T^S^_A_T^S^_C_ stacking using (**g**) GGA and (**h**) GGA + U method. Calculated unfolded band structure for (**f**) T_A_T_C_ and (**i**) T^S^_A_T^S^_C_ stacking using the GGA + U method.

Furthermore, we performed density functional theory (DFT) calculations to gain a comprehensive understanding of electronic structures in the above STS and ARPES experiments. A four-layer supercell with an ordered David-stars T_A_T_C_ stacking (Fig. 4a), identified by XRD superlattice reflections in 1T-TaS_2_ (Fig. 1f), was adopted. This configuration has been extensively employed in previous DFT calculations^13,14,20^. The absence of out-of-plane superlattice reflections in LC-TaS_2_ crystals indicates a disordered David-stars stacking configuration. Aiming to emphasize the influence of atomic layer sliding on the alteration of the electronic structures, T^S^_A_T^S^_C_ stacking (Fig. 4b), integrating David-stars T_A_T_C_ stacking with a translational shift representing disordered David-stars stacking due to broken translational symmetry along the *c*-direction, was adopted. The band structure for four layers 1T-TaS_2_ (Fig. 4d) with David-stars T_A_T_C_ stacking is in good agreement with previous reports^14,15,46^. However, for T^S^_A_T^S^_C_ stacking with a translational shift of 0.053 nm, the calculated band structures using generalized gradient approximation (GGA) exhibit metallic characteristics with a conduction band crossing the *E*_F_ at the L-point (Fig. 4g). To address this discrepancy, the Hubbard *U* term (GGA + U) is introduced in calculations, which opens a gap (Fig. 4h), confirming its Mott-insulator nature. For comparison purposes, the GGA + U method is also employed to calculate the band structure of T_A_T_C_ stacking, as shown in Fig. 4e. As expected, the limited increase in the energy gap after the introduction of the Hubbard *U* term indicates that on-site Coulomb repulsion is not the primary factor contributing to the energy gap for T_A_T_C_ stacking (Fig. 4c). In addition, interlayer distance contraction has negligible influence on the low-energy electronic structure beyond the reduced gap (Supplementary Fig. 9), confirming the dominant roles that laddering stack played in determining the interlayer coupling. The validity of simplifying the system to only 4 layers and employing a larger translational shift is discussed in the Methods part.

The density of states (DOS) represents gap opening induced by Mott-localization for the laddering stack structure (Fig. 4c). Although GGA calculations typically underestimate the gap size^13^, the DOS calculations show that the gap closes after laddering interlayer sliding, and reopens with a smaller value after introducing the Hubbard *U* term. This trend is consistent with the STS results (Fig. 2c).

The unfolded bands in the unit-cell Brillouin zone demonstrate the reduction in band dispersion along both Γ-A and Γ-M directions. Specifically, the dispersion of the flat band near −0.2 eV for T^S^_A_T^S^_C_ stacking (Fig. 4i) becomes flatter than that for T_A_T_C_ stacking (Fig. 4f). This is consistent with ARPES results at ~187 K, where the flat band centered at ~−0.2 eV for LC-TaS_2_ (Fig. 3e) is much narrower and less dispersive than that of 1T-TaS_2_ (Fig. 3b). In addition, the dispersion along the Γ-A direction is reduced to ~0.10 eV for T^S^_A_T^S^_C_ stacking from ~0.18 eV for T_A_T_C_ stacking. The reduced band dispersion aligns with observations from ARPES results. For LC-TaS_2_, the dominant peak at ~−0.2 eV exhibits less photon energy dependence (Fig. 3h).

The ARPES spectra, with a detected depth of 2–3 nm for electrons with 18 eV kinetic energy, extend across three layers^47^, providing multilayer information rather than characteristics of the top surface layer^22^. In LC-TaS_2_, the 2D flat bands close to *E*_F_ along both the Γ-A/*k*_z_ and Γ-M/*k*_//_ directions in the ARPES measurements and the wide energy gap determined from the STS spectra, in agreement with DFT calculations, confirms its 2D Mott-insulator nature. On the other hand, significant band dispersion in 1T-TaS_2_ implies its classification as a 3D band insulator^22^. It is noteworthy that, although DFT calculations based on T^S^_A_T^S^_C_ stacking generally capture the reduced band dispersion character in LC-TaS_2_, deviations in actual band dispersion values are present. This discrepancy may arise from alterations in the David-stars stacking sequence induced by atomic layer sliding, as illustrated in Supplementary Fig. 8, warranting further investigation.

### The effects of laddering interlayer sliding on electrical transport

**Fig. 5:**
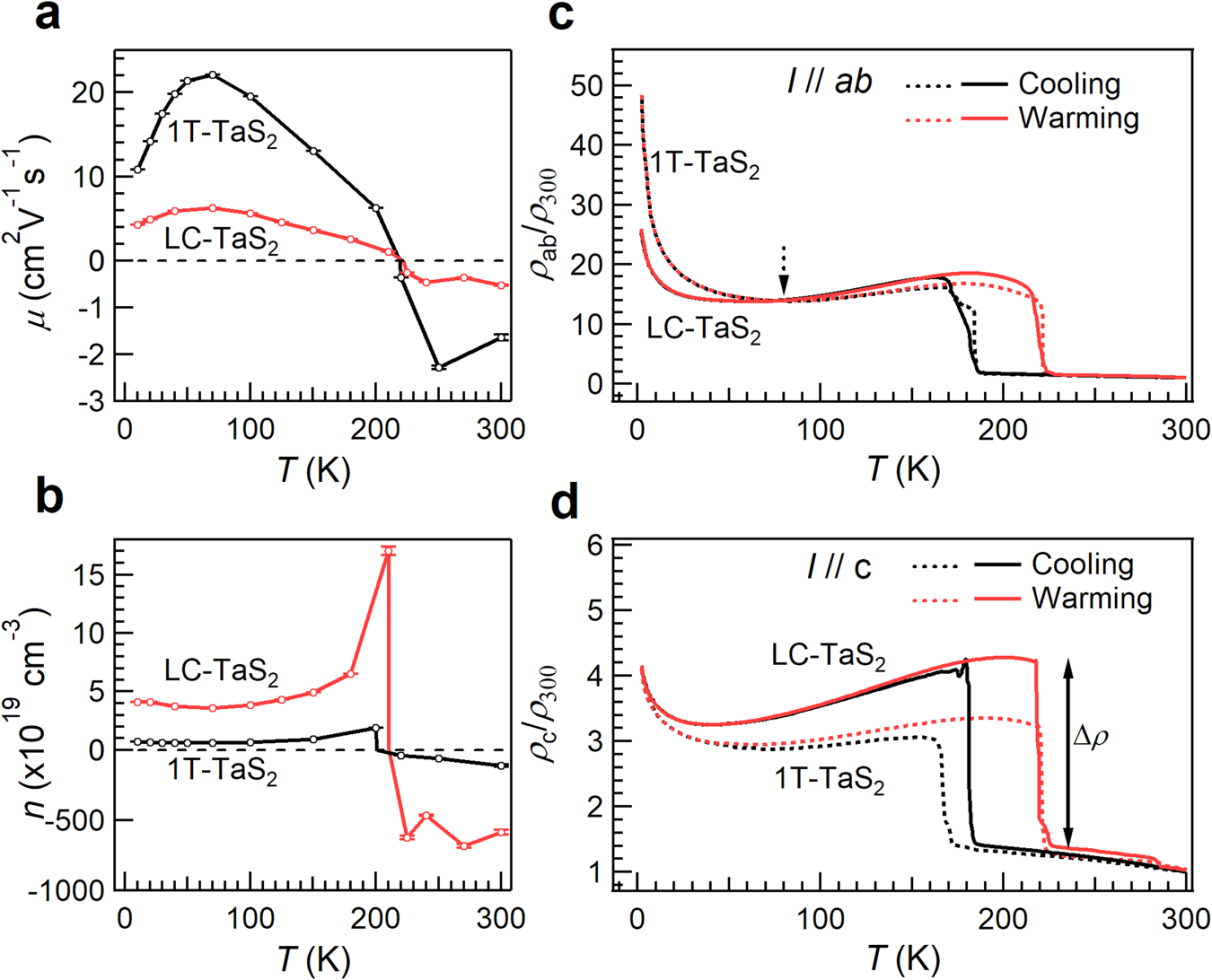
Anisotropic electrical transport properties. **a** The carrier mobility *μ* and (**b**) carrier concentrations *n* determined from the Hall measurements (see Supplementary Fig. 12). The negative values in panels (**a**, **b**) correspond to the electron concentration and electron mobility, respectively. The error bars are derived from the standard deviation of fitting for Hall resistivity, as shown in Supplementary Fig. 12d,e. Temperature dependence of (**c**) in-plane *ρ*_ab_/*ρ*_300_ and (**d**) out-of-plane *ρ*_c_/*ρ*_300_ for 1T-TaS_2_ and LC-TaS_2_ upon cooling and warming.

To explore the distinct macroscopic properties associated with this kind of duality of insulating states, anisotropic electrical transport measurements were conducted. Figure 5a, b show the temperature dependence of the mobility *μ* and carrier concentration *n*, respectively. The values were calculated using the formulas *n* = (e*R*_H_)^−1^ and *μ* = *R*_H_/*ρ*_xx_, where *ρ*_xx_ and *R*_H_ represent resistivity and Hall coefficient, respectively, as shown in Supplementary Fig. 12. The negative and positive signs denote the electron and hole carrier types, respectively. The obtained parameters for 1T-TaS_2_ are consistent well with the literature^48^. The sign change for both parameters corresponds to the transition from NC-CDW to C-CDW phase, as well as the gap opening observed in ARPES results (Fig. 3). In LC-TaS_2_, the electron localization and increased effective mass arising from the narrowing of flat bands, as revealed by ARPES, leads to a reduction in *μ* to ~0.3 times of the value of 1T-TaS_2_. Meanwhile, the ~6-fold increase in *n* for LC-TaS_2_ is dominated by the narrower Mott-gap, as revealed by STS, although the increase in the effective mass of carriers can offset part of the contribution. It is clear that the relatively large *μ* and small *n* observed in intrinsic 1T-TaS_2_ crystals, are conversed into the lowered *μ* and elevated *n* in LC-TaS_2_.

To better understand the underlying physics of laddering interlayer sliding, we normalized the resistivity to the values of NC-CDW state at 300 K (*ρ*_300 K_). This helps to exclude the effects of extrinsic factors and facilitate a comparison with 1T-TaS_2_ (Fig. 5c). Above 80 K, the resistivity behavior remains consistent even after laddering interlayer sliding, indicating unchanged in-plane David-stars configuration, although both *μ* and *n* are modulated by interlayer coupling. Below 80 K, the weaker insulating behavior of LC-TaS_2_ is consistent with its narrower energy gap.

For electrical transport between layers, resistivity with *I*//*c* was measured and plotted in Fig. 5d. The abrupt resistance jump (Δ*ρ*) induced by the first-order NC-to-C-CDW phase transition in *ρ*_c_/*ρ*_c(300 K)_ for LC-TaS_2_ is much larger than that for 1T-TaS_2_. This significant increase of Δ*ρ* can be attributed to the reduced band dispersion along *k*_z_ and, consequently, a larger effective mass of carriers in LC-TaS_2_ rising from the Mott-insulating mechanism.

## Discussion

Upon reviewing the aforementioned results, in T_A_ stacking (Fig. 1d), the David-stars with unpaired electrons dimerize to form bilayers with an even number of electrons^8,10,11,14–16,20–22,49^. Conversely, in the fractional translation of T^S^_A_ stacking (Fig. 1g), the non-dimerization of David-stars preserves an odd number of electrons within each David-star. This results in an enhancement of electron density within the David-star, consequently strengthening electron-electron interactions^9,19,20,50,51^. Here, the laddering stack structure is expected to gradually weaken the interlayer coupling by reducing the out-of-plane wavefunction overlap integral between David-stars of adjacent layers, thus reducing out-of-plane bandwidth *t*_⊥_. This reduction in *t*_⊥_ leads to an increase in Coulomb repulsion *U* due to the reduced electron screening. Consequently, the increased *U*/*t*_⊥_ ratio explains the switching from a band-insulator to a Mott-insulator mechanism^52^.

Our discovery introduces a translational degree of freedom for precise tuning of bulk crystal properties. It stands out for its simplicity, cleanliness, and seamless integration into device fabrication processes. This method opens up opportunities to investigate low-dimensional physics in layered 3D crystals^53^, expanding its applications beyond the realm of sliding ferroelectricity^28,29,32^.

## Methods

### Samples Preparation

High-quality 1T-TaS_2_ single crystals were grown by a chemical vapor transport (CVT) method^34,54,55^. First, tantalum and sulfur power with a 1:2 molar ratio were mixed and vacuum-sealed in the quartz tubes. After annealing at 800 °C for 3 days, polycrystalline TaS_2_ was obtained by quenching the quartz tubes in an ice-water mixture. Subsequently, the polycrystalline TaS_2_ powder and iodine (as a transport agent) were vacuum-sealed in quartz tubes. The quartz tubes were heated in a two-zone furnace for 10 days with different temperatures in the hot zone (850 °C) and cold zone (750 °C). The 1T-TaS_2_ crystals were obtained after quenching the quartz tubes in an ice-water mixture. The obtained 1T-TaS_2_ crystals were transferred to a vacuum chamber (better than 10^−2^ mbar) and annealed at 150–200 °C for 3–4 h. The LC-TaS_2_ crystals are obtained after cooling to room temperature naturally. All collected crystals were stored in an Ar-protected glove box before further characterization. Subsequently, the crystals were cut to pieces for electron microscopy and (synchrotron-based) XRD and PES characterization.

### Electron microscopy

HAADF-STEM images of the cross-sectional samples were collected at room temperature on an FEI Themis Z aberration-corrected scanning transmission electron microscope operating at 300 keV with a convergence angle of 30 mrad^56,57^.

### XRD measurement

The synchrotron-based XRD measurements were performed at the BL14B1 beamline of the Shanghai Synchrotron Radiation Facility (SSRF) using X-rays with a wavelength of *λ* = 1.24 Å and grazing incident angles of 1.0°. The corresponding spectra collected at room temperature are presented in scattering vector *q* coordinates by using the equation *q* = 4πsin*θ*/*λ*, where *θ* is half of the diffraction angle. The *q* has been calibrated by measuring the synchrotron-based XRD of a lanthanum hexaboride reference sample.

Lab-based XRD characterizations were performed on a Bruker D8 diffractometer using Cu K_α_ radiation to assess the quality of the obtained crystals. XRD data were also collected on 1T- and LC-TaS_2_ crystals after cooling down to 120 K.

### Synchrotron-based PES and ARPES

High-resolution PES was collected at the BL11U beamline of the National Synchrotron Radiation Laboratory (NSRL, China). The single crystals were cleaved and transferred into an ultra-high vacuum (UHV) chamber with a base pressure of 1 × 10^−10^ mbar. The S 2*p* and Ta 4*f* spectra were measured at normal emission and room temperature using 240 eV photon energy. The photon energy was calibrated using the Au 4*f*_7/2_ core-level peak (84.0 eV) of a gold foil in electrical contact with the samples. The least-squares peak fitting was performed employing a Shirley background and asymmetric peak profiles for TaS_2_ species.

The ARPES measurements were performed at the BL13U beamline of NSRL. The single crystals, previously qualified by XRD, were cooled to 280 K, cleaved, and measured in situ in a UHV chamber with a base pressure of 1 × 10^−10^ mbar. The crystals were further cooled to a selective temperature and maintained for 10 min before measurement. The Fermi energy was referred to as a gold foil in electrical contact with the samples.

### STM/STS measurement

The STM/STS measurements were conducted in an Omicron LT-STM system. The obtained 1T- and LC-TaS_2_ single crystals, qualified by XRD, were cleaved and transferred into a UHV prep-chamber with a base pressure of 5 × 10^−10^ mbar. After degassing at approximately 100 °C for 1 h, the distinct samples were transferred to the analysis chamber with a base pressure of 1 × 10^−10^ mbar for STM/STS measurements. STM topography images were collected in constant-current mode. STS measurements were performed in constant-height mode using a standard lock-in technique (*f* = 773 Hz, *V*_r.m.s._ = 15 mV). The STM tip was calibrated spectroscopically on the Au(111) surface. All data were collected at 4.5 K.

### Transport measurement and sources of error in data fitting

The electrical transport measurements for freshly cleaved single crystals, cut from XRD-qualified samples, were carried out by using a physical property measurement system (PPMS, Quantum Design) with a standard six-probe method. The warming and cooling rates are 3 K/min. The in-plane and out-of-plane resistivity data for LC-TaS_2_ are collected on two identical samples cut from one single crystal.

While linear fittings to Hall resistivity provide satisfactory descriptions of experimental data (Supplementary Fig. 12d, e), standard fitting errors persist. These errors subsequently introduced inaccuracies in related parameters, such as *μ* and *n* (Fig. 5a, b). Notably, the standard fitting errors for Hall coefficient were four orders of magnitudes smaller than the obtained parameters, ensuring the high reliability of both the fitting process and its related outcomes.

### DFT calculation

The first-principles calculations based on DFT were performed using the Vienna Ab initio Simulation Package (VASP)^58,59^. The pseudopotential was described using the projector augmented wave (PAW) methods, and the exchange-correlation interaction was treated using GGA, which is parameterized by Perdew-Burke-Ernzerhof (PBE)^60^. The on-site Coulomb repulsion *U* = 2.94 eV, previously established using a self-consistent method^61^, was included for the tantalum 5*d* orbitals. The energy cutoff for the plane wave was set to 400 eV. The van der Waals interaction was accounted for using Grimme’s DFT-D3 of the semi-empirical method^62^. Brillouin zone sampling was performed using a 5 × 5 × 5 *k*-point mesh for electronic properties calculation. The band unfolding was processed using the VASPKIT code^63^.

To examine the influence of laddering interlayer sliding on the electronic structure, first-principles calculations were performed. The initial assumption was made for pristine 1T-TaS_2_ crystals, which were considered to exhibit an out-of-plane paired T_A_T_C_ David-stars stacking sequence (Fig. 4a). This specific stacking arrangement, characterized by stacking vectors of **T**_A_ = **c** and **T**_C_ = 2**a** + **c**, has been proposed in previous experimental studies and was employed in DFT calculations^13,14,20^. To simulate the interlayer sliding effect, the corresponding T^S^_A_T^S^_C_ stacking configuration was considered (Fig. 4e). In this configuration, **T**^S^_A_ = δ**a** + **c** and **T**^S^_C_ = (2+δ)**a** + **c**, with δ**a** representing the lateral sliding distance.

Simulating the experimentally observed 40-layer supercell structure, which contains 1560 Ta- and S-atoms, poses challenges. As a result, a simplified structure with a minimum of 4 layers or one T^S^_A_T^S^_C_ stacking period was adopted. To compensate for the effects of this structural simplification, a larger sliding distance δ**a** of 0.053 nm (1/6**a**) was used. The validity of this approach was verified by varying δ**a** from 0.039 nm (1/8**a**) to 0.106 nm (1/3**a**). Supplementary Fig. 10 demonstrates that the band structures calculated with δ**a** ≥ 0.053 nm (1/6**a**) accurately reproduce results from the four-layer supercell structure. To clarify the coordination effect between supercell layer numbers and sliding distance in representing experimental observation, extended calculations of eight-layer supercell structures were performed. Supplementary Fig. 11 illustrates the corresponding band structures for various δ**a**. Notably, a 0.039 nm (1/8**a**) value is identified to close the gap for a larger eight-layer supercell structure, which is smaller than 0.053 nm (1/6**a**) for the four-layer supercell structure (Supplementary Fig. 11e). The observations lead to the conclusion that a sliding distance ≤√3/*n***a** can effectively close the gap for an *n*-layer supercell structure (Supplementary Fig. 11f). Therefore, it is reasonable to propose that √3/40**a** sliding can close the gap in a 40-layer supercell structure, as observed experimentally.

Notably, the GGA + U method has limitations regarding the accuracy of gap value in this system, given its sensitivity to the on-site *U* value. A potential alternative approach is the DFT + GOU method, where the self-consistent $\bar{U}$ acts on the entire David-star cluster. This method may offer a more precise description of the electronic structure^19,42^, warranting further theoretical investigation.

### Supplementary information

Supplementary Information

Peer Review File

SourceData

## Data Availability

All data generated in this study are provided in the Article and the Supplementary Information. Additional data related to this work are available from the corresponding author upon request. Source data are provided with this paper.

## References

[1] Lee PA, Nagaosa N, Wen X-G (2006). Doping a Mott insulator: physics of high-temperature superconductivity. Rev. Mod. Phys..

[2] Neupert T, Denner MM, Yin J-X, Thomale R, Hasan MZ (2022). Charge order and superconductivity in kagome materials. Nat. Phys..

[3] Nie L (2022). Charge-density-wave-driven electronic nematicity in a kagome superconductor. Nature.

[4] Persky E (2022). Magnetic memory and spontaneous vortices in a van der Waals superconductor. Nature.

[5] Fazekas P, Tosatti E (1979). Electrical, structural and magnetic properties of pure and doped 1T-TaS2. Philos. Mag. B.

[6] Sipos B (2008). From Mott state to superconductivity in 1T-TaS_2_. Nat. Mater..

[7] Dong Q (2021). Structural phase transition and superconductivity hierarchy in 1T-TaS_2_ under pressure up to 100 GPa. npj Quantum Mater..

[8] Ma L (2016). A metallic mosaic phase and the origin of Mott-insulating state in 1T-TaS_2_. Nat. Commun..

[9] Cho D (2016). Nanoscale manipulation of the Mott insulating state coupled to charge order in 1T-TaS_2_. Nat. Commun..

[10] Darancet P, Millis AJ, Marianetti CA (2014). Three-dimensional metallic and two-dimensional insulating behavior in octahedral tantalum dichalcogenides. Phys. Rev. B.

[11] Wen C (2021). Roles of the narrow electronic band near the fermi level in 1T-TaS_2_-telated layered materials. Phys. Rev. Lett..

[12] Vaňo V (2021). Artificial heavy fermions in a van der Waals heterostructure. Nature.

[13] Petocchi F (2022). Mott versus hybridization gap in the low-temperature phase of 1T*-*TaS_2_. Phys. Rev. Lett..

[14] Lee S-H, Goh JS, Cho D (2019). Origin of the insulating phase and first-order metal-insulator transition in 1T-TaS_2_. Phys. Rev. Lett..

[15] Ritschel T (2015). Orbital textures and charge density waves in transition metal dichalcogenides. Nat. Phys..

[16] Ritschel T, Berger H, Geck J (2018). Stacking-driven gap formation in layered 1T-TaS_2_. Phys. Rev. B.

[17] Lee J, Jin K-H, Yeom HW (2021). Distinguishing a Mott Insulator from a trivial insulator with atomic adsorbates. Phys. Rev. Lett..

[18] Li C-K, Yao X-P, Liu J, Chen G (2022). Fractionalization on the surface: Is type-II terminated 1T-TaS_2_ surface an anomalously realized spin liquid?. Phys. Rev. Lett..

[19] Shin D (2021). Identification of the Mott insulating charge density wave state in 1T-TaS_2_. Phys. Rev. Lett..

[20] Butler CJ, Yoshida M, Hanaguri T, Iwasa Y (2020). Mottness versus unit-cell doubling as the driver of the insulating state in 1T-TaS_2_. Nat. Commun..

[21] Wu Z (2022). Effect of stacking order on the electronic state of 1T-TaS_2_. Phys. Rev. B.

[22] Wang YD (2020). Band insulator to Mott insulator transition in 1T-TaS_2_. Nat. Commun..

[23] Martino E (2020). Preferential out-of-plane conduction and quasi-one-dimensional electronic states in layered 1T-TaS_2_. npj 2D Mater. Appl..

[24] Klanjšek M (2017). A high-temperature quantum spin liquid with polaron spins. Nat. Phys..

[25] Kratochvilova M (2017). The low-temperature highly correlated quantum phase in the charge-density-wave 1T-TaS_2_ compound. npj Quantum Mater..

[26] Mañas-Valero S, Huddart BM, Lancaster T, Coronado E, Pratt FL (2021). Quantum phases and spin liquid properties of 1T-TaS_2_. npj Quantum Mater..

[27] Law KT, Lee PA (2017). 1T-TaS_2_ as a quantum spin liquid. Proc. Natl Acad. Sci. USA.

[28] Yasuda K, Wang X, Watanabe K, Taniguchi T, Jarillo-Herrero P (2021). Stacking-engineered ferroelectricity in bilayer boron nitride. Science.

[29] Vizner Stern M (2021). Interfacial ferroelectricity by van der Waals sliding. Science.

[30] Wang X (2022). Interfacial ferroelectricity in rhombohedral-stacked bilayer transition metal dichalcogenides. Nat. Nanotechnol..

[31] Wu M, Li J (2021). Sliding ferroelectricity in 2D van der Waals materials: related physics and future opportunities. Proc. Natl Acad. Sci. USA.

[32] Deb S (2022). Cumulative polarization in conductive interfacial ferroelectrics. Nature.

[33] Hovden R (2016). Atomic lattice disorder in charge-density-wave phases of exfoliated dichalcogenides (1T-TaS_2_). Proc. Natl Acad. Sci. USA.

[34] Liu Y (2013). Superconductivity induced by Se-doping in layered charge-density-wave system 1T-TaS_2−x_Se_x_. Appl. Phys. Lett..

[35] Eda G (2012). Coherent atomic and electronic heterostructures of single-layer MoS_2_. ACS Nano.

[36] Yin X (2017). Tunable inverted gap in monolayer quasi-metallic MoS2 induced by strong charge-lattice coupling. Nat. Commun..

[37] Pollak RA, Hughes HP (1976). Charge density density wave phase transitions observed by X-ray photoemission. J. Phys. Colloq..

[38] Hughes HP, Scarfe JA (1995). Site specific photohole screening in a charge density wave. Phys. Rev. Lett..

[39] Wang Z (2018). Surface-limited superconducting phase transition on 1T-TaS_2_. ACS Nano.

[40] Zhu X-Y (2019). Realization of a metallic state in 1T-TaS_2_ with persisting long-range order of a charge density wave. Phys. Rev. Lett..

[41] Lin H (2020). Scanning tunneling spectroscopic study of monolayer 1T-TaS_2_ and 1T-TaSe_2_. Nano Res..

[42] Dong J (2023). Electronic dispersion, correlations and stacking in the photoexcited state of 1T-TaS2. 2D Mater..

[43] Yu Y (2015). Gate-tunable phase transitions in thin flakes of 1T-TaS_2_. Nat. Nanotechnol..

[44] Dong J (2023). Dynamics of electronic states in the insulating intermediate surface phase of 1T-TaS_2_. Phys. Rev. B.

[45] Cha S (2023). Order-disorder phase transition driven by interlayer sliding in lead iodides. Nat. Commun..

[46] Zhang W, Wu J (2023). Stacking order and driving forces in the layered charge density wave phase of 1T-MX_2_ (M = Nb, Ta and X = S, Se). Mater. Res. Express.

[47] Seah MP, Dench WA (1979). Quantitative electron spectroscopy of surfaces: a standard data base for electron inelastic mean free paths in solids. Surf. Interface Anal..

[48] Inada R, Ōnuki Y, Tanuma S (1979). Hall effect of 1T-TaS_2_. Phys. Lett. A.

[49] Stahl Q (2020). Collapse of layer dimerization in the photo-induced hidden state of 1T-TaS_2_. Nat. Commun..

[50] Kim J-J, Yamaguchi W, Hasegawa T, Kitazawa K (1994). Observation of Mott localization gap using low temperature scanning tunneling spectroscopy in commensurate 1*T*-TaS_2_. Phys. Rev. Lett..

[51] Cho D, Cho Y-H, Cheong S-W, Kim K-S, Yeom HW (2015). Interplay of electron-electron and electron-phonon interactions in the low-temperature phase of 1T-TaS_2_. Phys. Rev. B.

[52] Kancharla SS, Okamoto S (2007). Band insulator to Mott insulator transition in a bilayer Hubbard model. Phys. Rev. B.

[53] Wang J, Cheng F, Sun Y, Xu H, Cao L (2024). Stacking engineering in layered homostructures: transitioning from 2D to 3D architectures. Phys. Chem. Chem. Phys..

[54] Bu K (2019). Possible strain induced Mott gap collapse in 1T-TaS_2_. Commun. Phys..

[55] Shen S (2020). Single-water-dipole-layer-driven reversible charge order transition in 1T-TaS_2_. Nano Lett..

[56] Wang W (2022). Electrical manipulation of skyrmions in a chiral magnet. Nat. Commun..

[57] Tang J (2021). Magnetic skyrmion bundles and their current-driven dynamics. Nat. Nanotechnol..

[58] Kresse G, Furthmüller J (1996). Efficient iterative schemes for ab initio total-energy calculations using a plane-wave basis set. Phys. Rev. B.

[59] Kresse G, Joubert D (1999). From ultrasoft pseudopotentials to the projector augmented-wave method. Phys. Rev. B.

[60] Perdew JP, Burke K, Ernzerhof M (1996). Generalized gradient approximation made simple. Phys. Rev. Lett..

[61] Boix-Constant C (2021). Out-of-plane transport of 1T-TaS_2_/graphene-based van der Waals heterostructures. ACS Nano.

[62] Grimme S, Ehrlich S, Goerigk L (2011). Effect of the damping function in dispersion corrected density functional theory. J. Comput. Chem..

[63] Wang V, Xu N, Liu J-C, Tang G, Geng W-T (2021). VASPKIT: a user-friendly interface facilitating high-throughput computing and analysis using VASP code. Comput. Phys. Commun..

