# Supplementary material for: Dualistic insulator states in 1T-TaS2 crystals
Source: Nat Commun. 2024 Apr 23;15:3425. doi: 10.1038/s41467-024-47728-0 (PMC11039681; doi:10.1038/s41467-024-47728-0)
Supplement: Supplementary file 1 — Supplementary Information [file 41467_2024_47728_MOESM1_ESM.pdf]

Supplementary Information:

Dualistic insulator states in 1T-TaS_2 crystals

Yihao Wang[1,†], Zhihao Li[1,2†], Xuan Luo[3], Jingjing Gao[3], Yuyan Han[1], Jialiang Jiang[1], Jin Tang[4], Huanxin Ju[5], Tongrui Li[6], Run Lv[3,7], Shengtao Cui[6], Yingguo Yang[8], Yuping Sun[1,3,9], Junfa Zhu[6], Xingyu Gao[10], Wenjian Lu[3,*], Zhe Sun[6,9,11,*], Hai Xu[2,12,*], Yimin Xiong[4,11,*], Liang Cao[1,*]

[1]Anhui Key Laboratory of Low-Energy Quantum Materials and Devices, High Magnetic Field Laboratory, HFIPS, Chinese Academy of Sciences, Hefei 230031, P. R. China

[2]Changchun Institute of Optics, Fine Mechanics and Physics, Chinese Academy of Sciences, Changchun, Jilin 130033, P. R. China

[3]Key Laboratory of Materials Physics, Institute of Solid State Physics, HFIPS, Chinese Academy of Sciences, Hefei 230031, P. R. China

[4]Department of Physics, School of Physics and Optoelectronics Engineering, Anhui University, Hefei 230601, P. R. China

[5]PHI Analytical Laboratory, ULVAC-PHI Instruments Co., Ltd., Nanjing 211110, Jiangsu, P. R. China

[6]National Synchrotron Radiation Laboratory, University of Science and Technology of China, Hefei 230026, P. R. China

[7]Science Island Branch of Graduate School, University of Science and Technology of China, Hefei 230026, P. R. China

[8]State Key Laboratory of Photovoltaic Science and Technology, School of Microelectronics, Fudan University, Shanghai 200433, P. R. China

[9]Collaborative Innovation Center of Advanced Microstructures, Nanjing University, Nanjing 210093, P. R. China

[10]Shanghai Synchrotron Radiation Facility (SSRF), Zhangjiang Laboratory, Shanghai Advanced Research Institute, Chinese Academy of Sciences, 239 Zhangheng Road, Shanghai 201204, P. R. China

[11]Hefei National Laboratory, Hefei 230028, P. R. China

[12]Center of Materials Science and Optoelectronics Engineering, University of Chinese Academy of Sciences, Beijing 100049, P. R. China

*Author to whom correspondence should be addressed: wjlu@issp.ac.cn (W. L.) zsun@ustc.edu.cn (Z. S.); xuhai@ciomp.ac.cn (H. X.); yxiong@ahu.edu.cn (Y. X.); lcao@hmfl.ac.cn (L. C.)

† Y.W. and Z.L. contributed equally to this work.

Supplementary Note 1: High-angle annular dark field (HAADF) scanning transmission electron microscopy (STEM) images

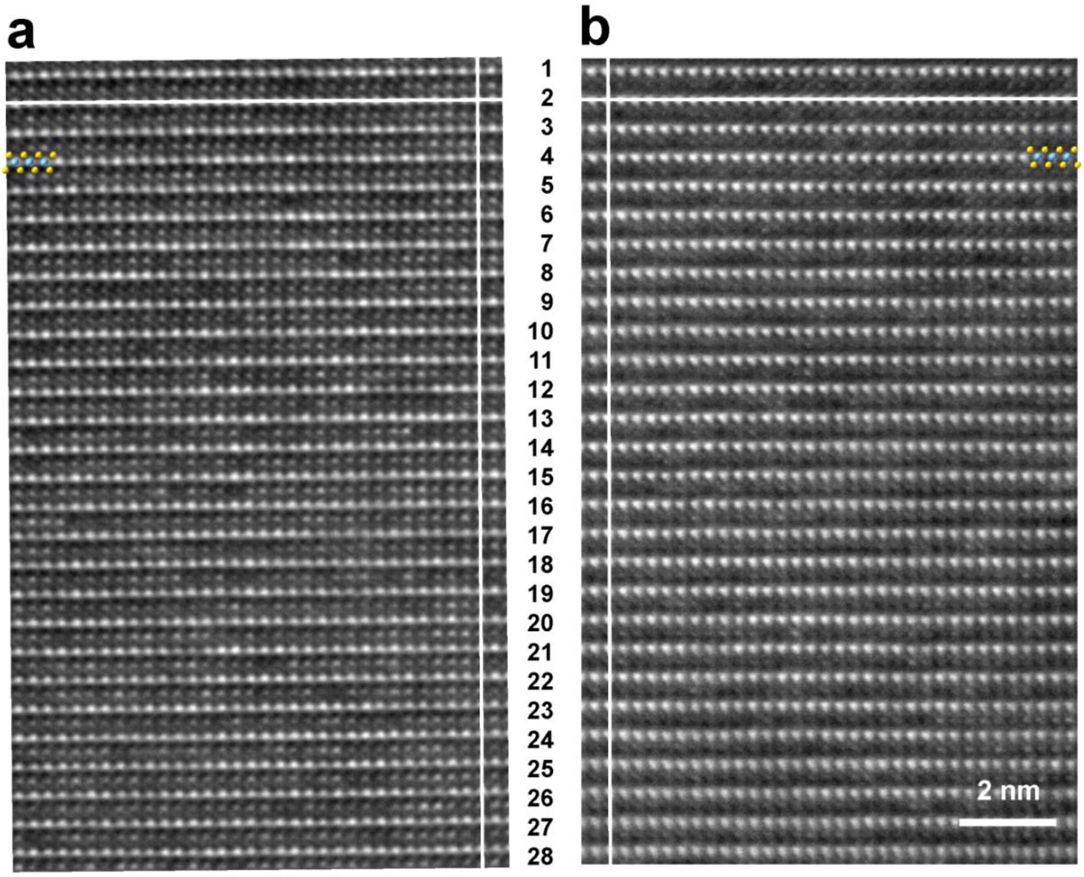

Supplementary Fig. 1. Vertical aligned atomic stack structure. The cross-sectional HAADF-STEM images for 1T-TaS_2 crystals collected along the **a** [100] and **b** [110] directions.

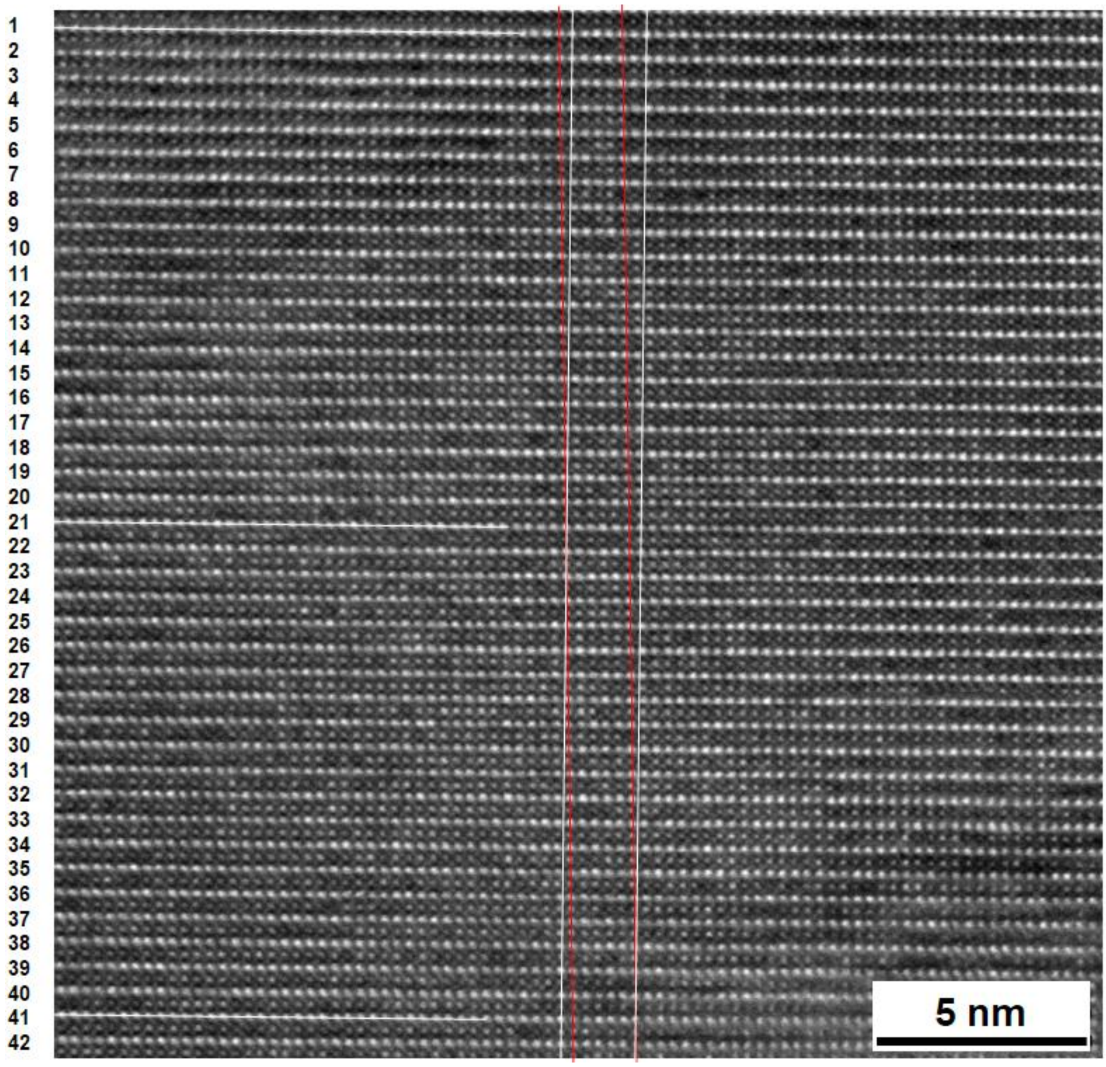

Supplementary Fig. 2. Laddering stack structure. The cross-sectional HAADF-STEM image for LC-TaS_2 crystals collected along the [100] direction. The white horizontal and vertical solid lines indicate the in-plane and out-of-plane direction, respectively. The red solid lines highlight the atomic misalignment.

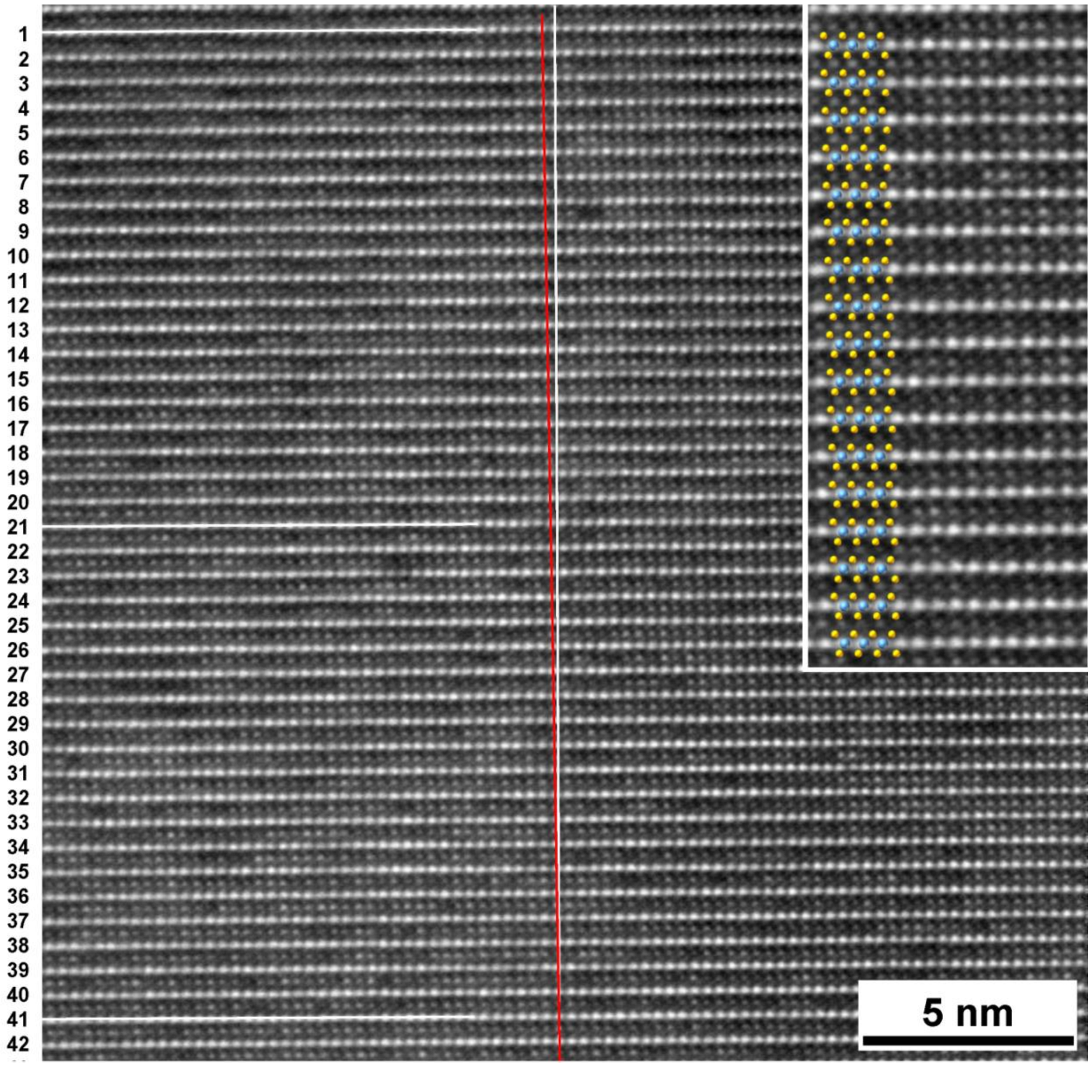

Supplementary Fig. 3. Laddering stack structure. The cross-sectional HAADF-STEM image for LC-TaS_2 crystals collected along the [110] direction.

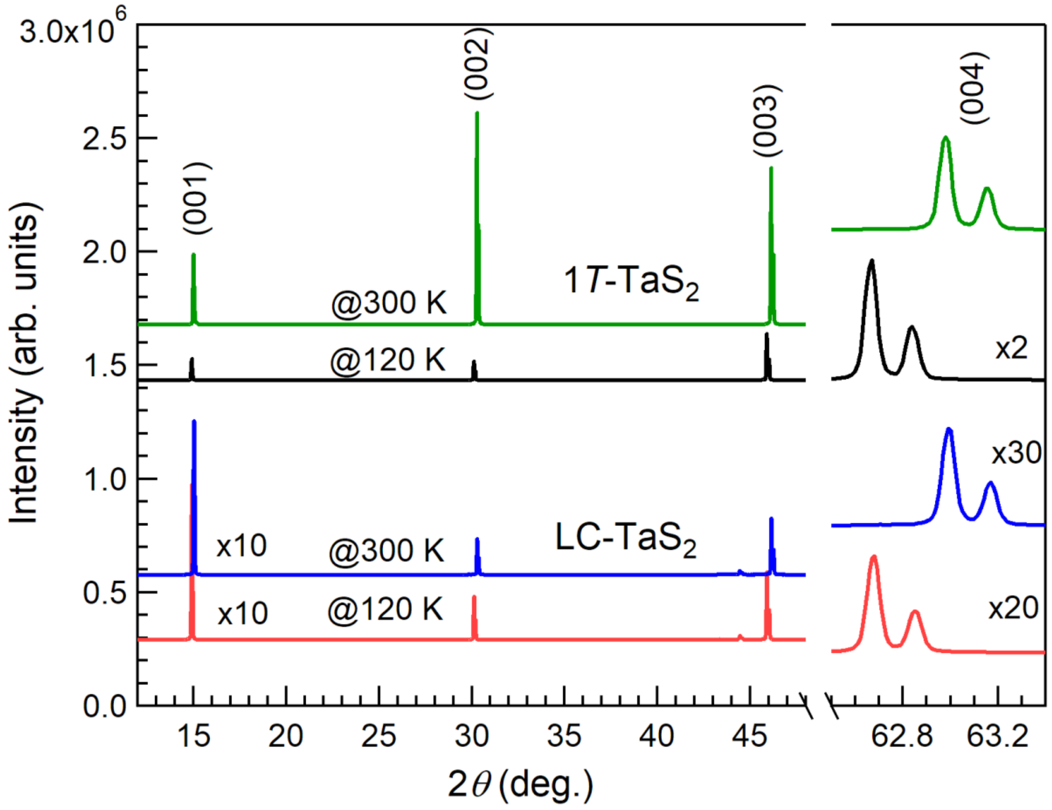

Supplementary Fig. 4. Temperature dependent XRD characterization. XRD patterns for 1T-TaS_2 and LC-TaS_2 crystals measured at 120 K and 300 K, respectively.

Supplementary Note 2: Photoemission spectroscopy

The A-Ta, B-Ta and C-Ta features are attributed to the one central Ta-atom at A-site, six nearest neighbor Ta-atoms at B-site, six next-nearest neighbor Ta-atoms at C-cite, respectively, within the David stars (Fig. 1b in the main text). The lower overlap of B-Ta and A-Ta features leads to a decrease in the maximum intensity at the B-Ta feature position, which is also self-consistent with a constant intensity ratio of 6(C-Ta):6(B-Ta):1(A-Ta).

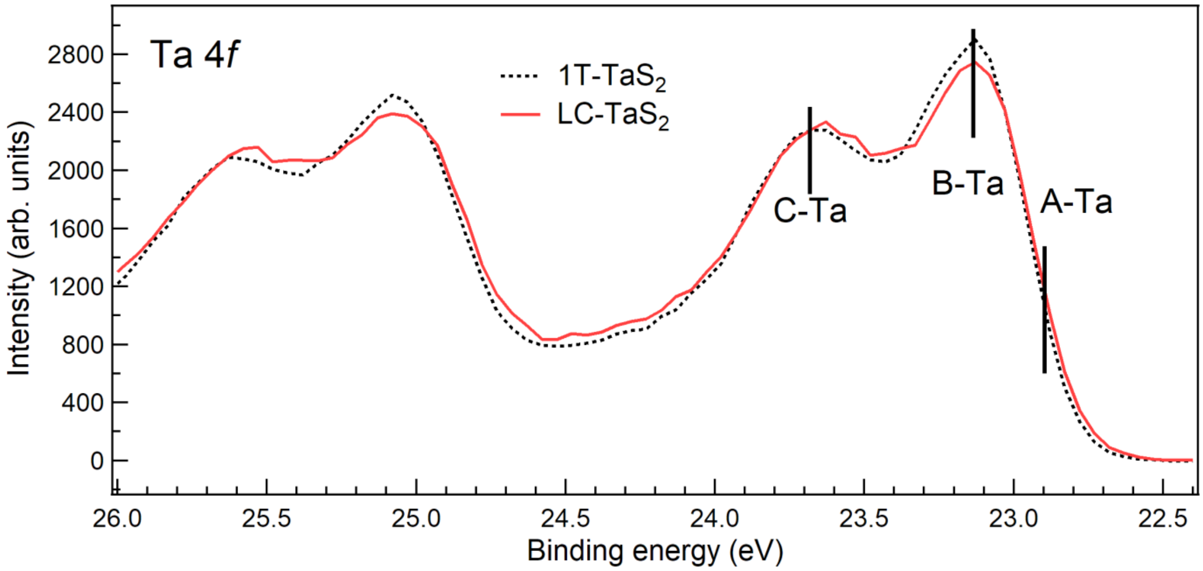

Supplementary Fig. 5. Charge re-distribution induced chemical shift. Ta 4*f* core level spectra for 1T-TaS_2 and LC-TaS_2 measured at room temperature using photon energy of 240 eV.

Supplementary Note 3: Scanning tunneling microscopy/spectroscopy (STM/STS)

Assuming the lower layer maintains uniform CDW domain patterns, configurations of both T_A^S-stacking (guided by black dots) and T_C^S-stacking (guided by blue and black dots) configurations are proposed in Supplementary Fig. 6**a**.

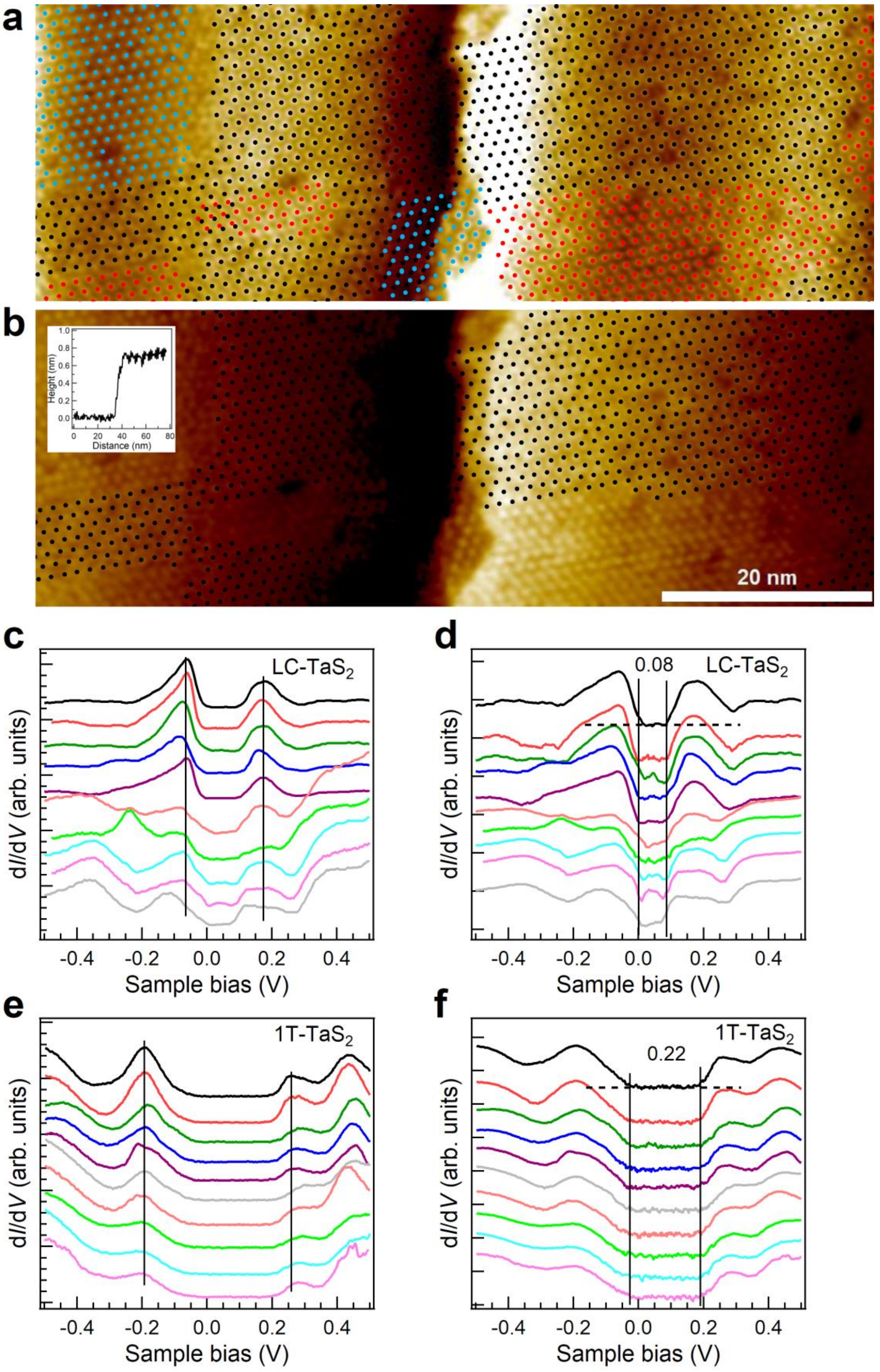

Supplementary Fig. 6. Surface CDW stacking configuration and repeatability of STS curves. a The STM topography of the step area for LC-TaS_2. **b** The same image with lower

contrast to highlight the CDW domain pattern in upper layer. The inset shows a height profile across the step. The color dots in panel **a** and **b** guide the David-stars centers. A series of d*I*/d*V* spectra with **c,e** linear intensity scale and **d,f** logarithmic intensity scale measured at different surface locations of LC-TaS_2 and 1T-TaS_2 crystals, respectively.

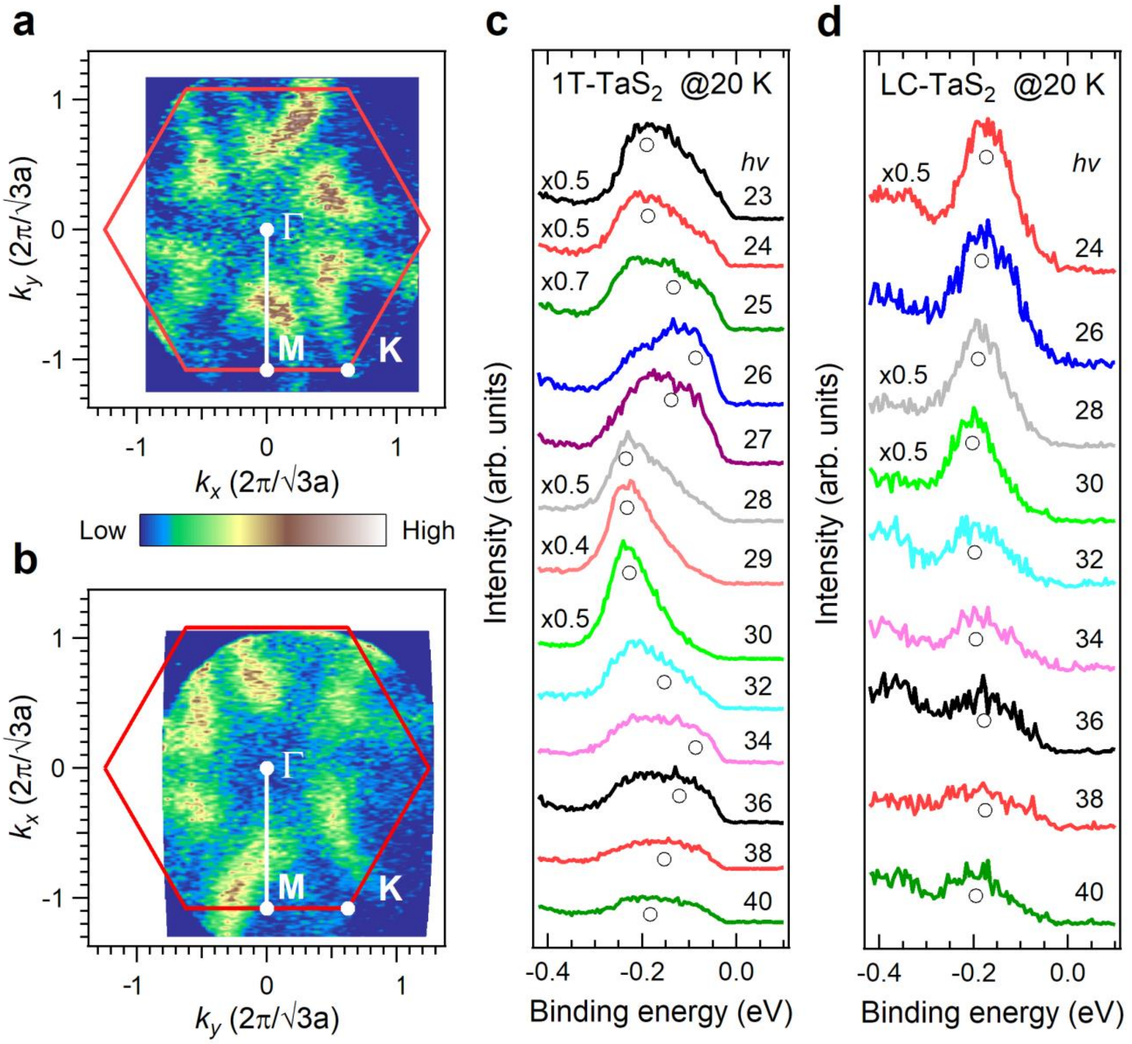

Supplementary Fig. 7. ARPES mapping and band dispersions along the k_z direction. The ARPES constant energy maps measured at 0.2 eV below Fermi level and at 280 K for **a** 1T-TaS_2 and **b** LC-TaS_2, respectively. Photon energy dependence of energy distribution curves (EDCs) in the insulating phase collected at Γ-point and 20 K for **c** 1T-TaS_2 and **d** LC-TaS_2, respectively.

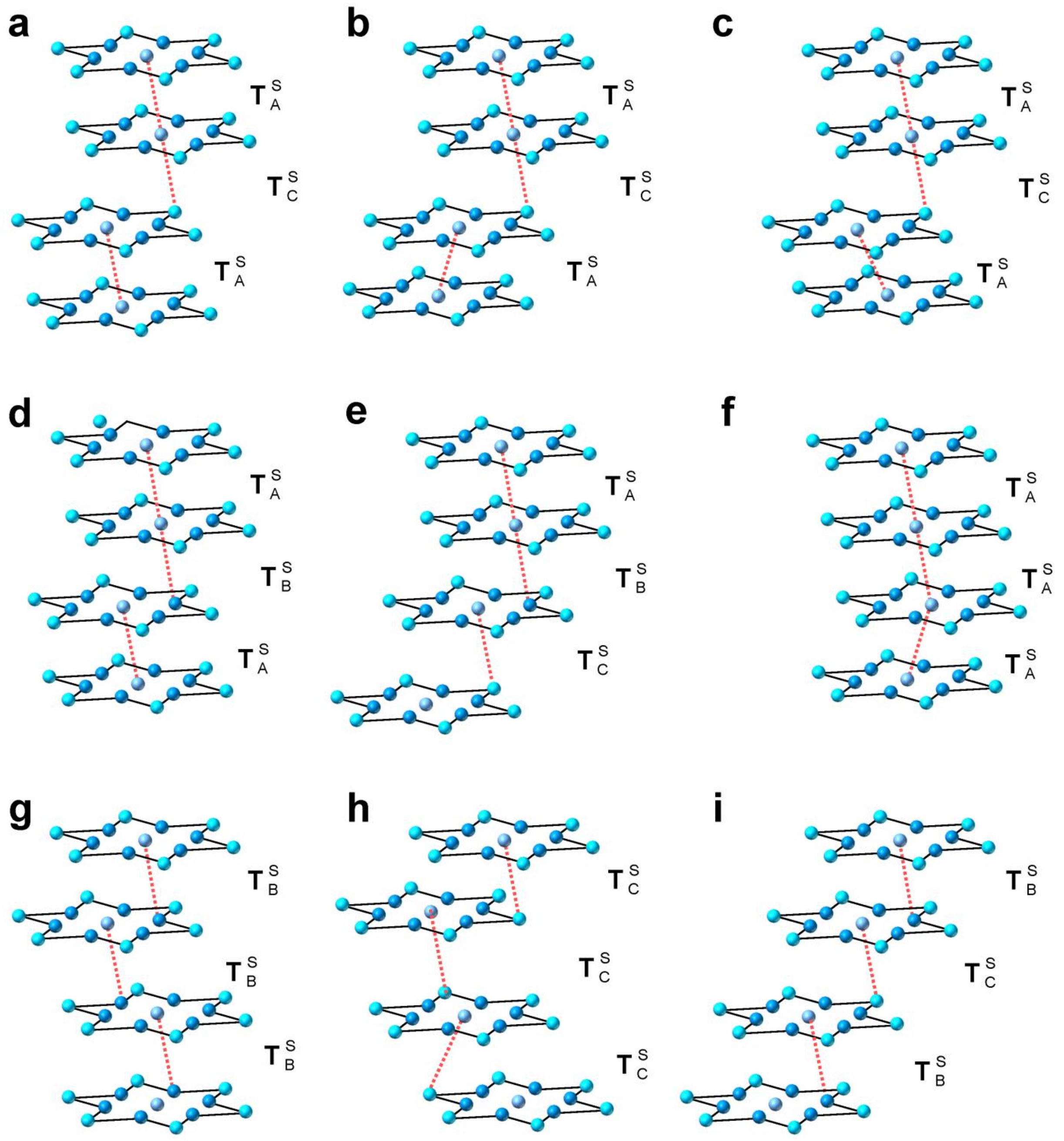

Supplementary Fig. 8. Potential configurations of disordered David-stars stacking in 4-layer supercells. **a** $T_A^S T_C^S$ and **b,c** its random stacking counterpart, **d** $T_A^S T_B^S$, **e** $T_A^S T_B^S T_C^S$, **f** T_A^S, **g** T_B^S, **h** T_C^S, and **i** $T_B^S T_C^S$. Note that **g-h** do not contain T_A^S stacking observed in STM image (Supplementary Fig. 6).

Supplementary Note 4: Density functional theory (DFT) calculation.

To clarify the dominate roles that *c*-lattice contraction and in-plane shift play in determining the interlayer coupling, band structures were calculated with reduced *c*-lattice parameter. Supplementary Fig. 9 displays the band structures for T_AT_C stacking with δ**a**=0.53 Å. c_0 represents the theoretically optimized lattice parameter of the T_AT_C stacking. Remarkably, despite these structural adjustments, the low-energy electronic structure exhibited negligible changes. GGA calculations reveal that the electron pocket at the L-point experiences subtle enlargement with reduced *c*-lattice parameter, whereas the whole band dispersion remains almost unchanged. GGA+U calculation results demonstrate that as the *c*-lattice parameter decreases, the energy gap decreases slightly.

To clarify the coordination effect between supercell layer numbers and sliding distance in representing experimental electronic structures, we expanded our calculations to 8-layer supercell structures without Hubbard *U*. Supplementary Fig 11 illustrates the corresponding band structures for multiple values of in-plane displacement. Supplementary Fig. 11e summarized the evolution of calculated energy gap as a function of in-plane displacement for both 4- and 8-layer supercell. Without considering Hubbard *U*, the energy gap gradually decreases as the sliding distance increases and closes for δ**a** exceeding 0.32 Å (1/10**a**). Notably, a 0.32 Å (1/10**a**) value is found to close the gap for a larger 8-layer supercell structure, which is smaller than 0.53 Å (1/6**a**) required for the 4-layer supercell structure. Supplementary Fig. 11f represents the evolution of in-plane displacement √3/*n***a** for *n*-layer supercell structures. The in-plane displacements that could close the gap, as extracted from calculations, are consistently smaller than √3/*n***a**. We thus conclude that ≤√3/*n***a** in-plane displacement can close the gap for an *n*-layer supercell structure.

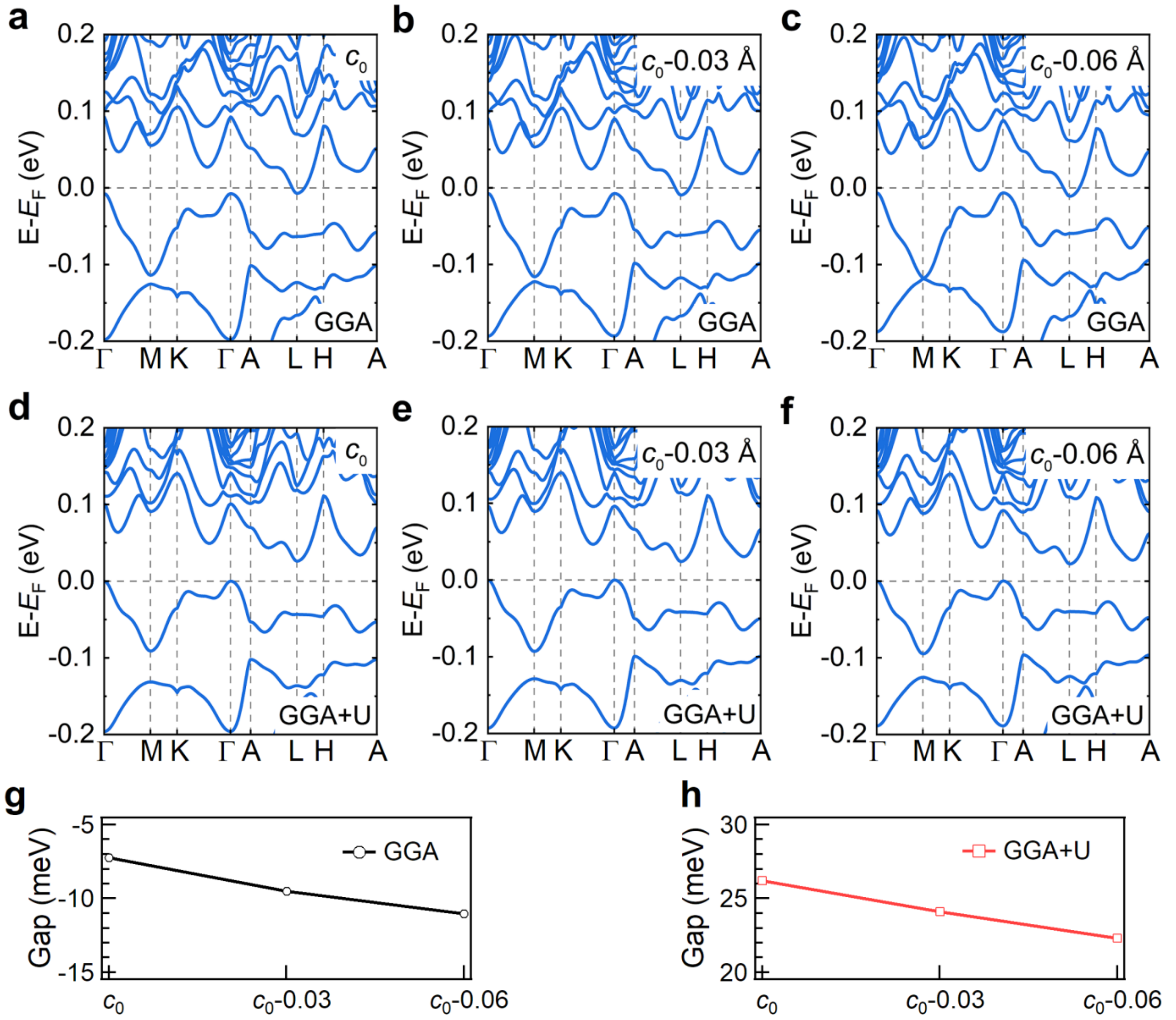

Supplementary Fig. 9. DFT calculated band structures with reduced *c*-lattice parameter. Band structure for T_AT_C stacking with reduced *c*-lattice parameter of **a** c_0, **b** c_0-0.03 Å, and **c** c_0-0.06 Å, respectively, using GGA. c_0 represents the theoretically optimized lattice parameter of the T_AT_C stacking. **d-f** Corresponding results calculated using GGA+U. The gap values for **g** GGA and **h** GGA+U calculations determined by the difference of the valence band maximum (VBM) and conduction band minimum (CBM). The negative gap indicates a metal state.

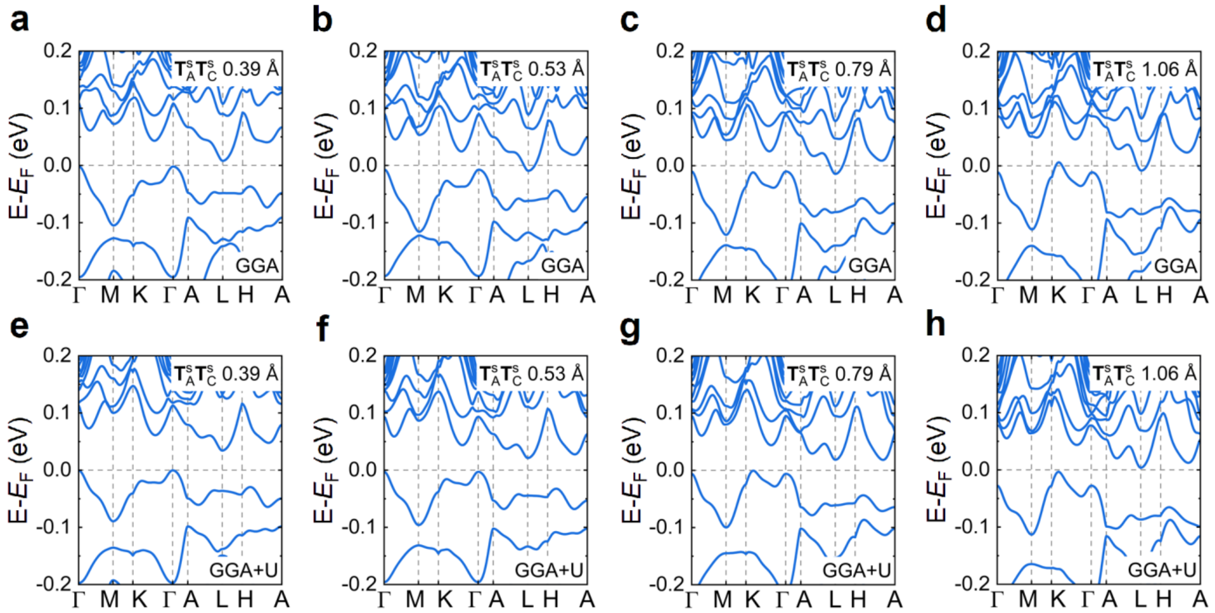

Supplementary Fig. 10. DFT calculated band structures of 4-layer supercell structures. Band structure for $T_A^S T_C^S$ stacking with different sliding distances δ**a** of **a** 0.39Å (1/8**a**), **b** 0.53Å (1/6**a**), **c** 0.76Å (1/4**a**), and **d** 1.06Å (1/3**a**) without Hubbard *U*, and **e-h** with Hubbard *U*, respectively. Without considering Hubbard *U*, the energy gap gradually decreases as the sliding distance increases and closes for δ**a** exceeding 0.53 Å (1/6**a**). The introduction of *U* leads to the reopening of energy gap for all δ**a** values.

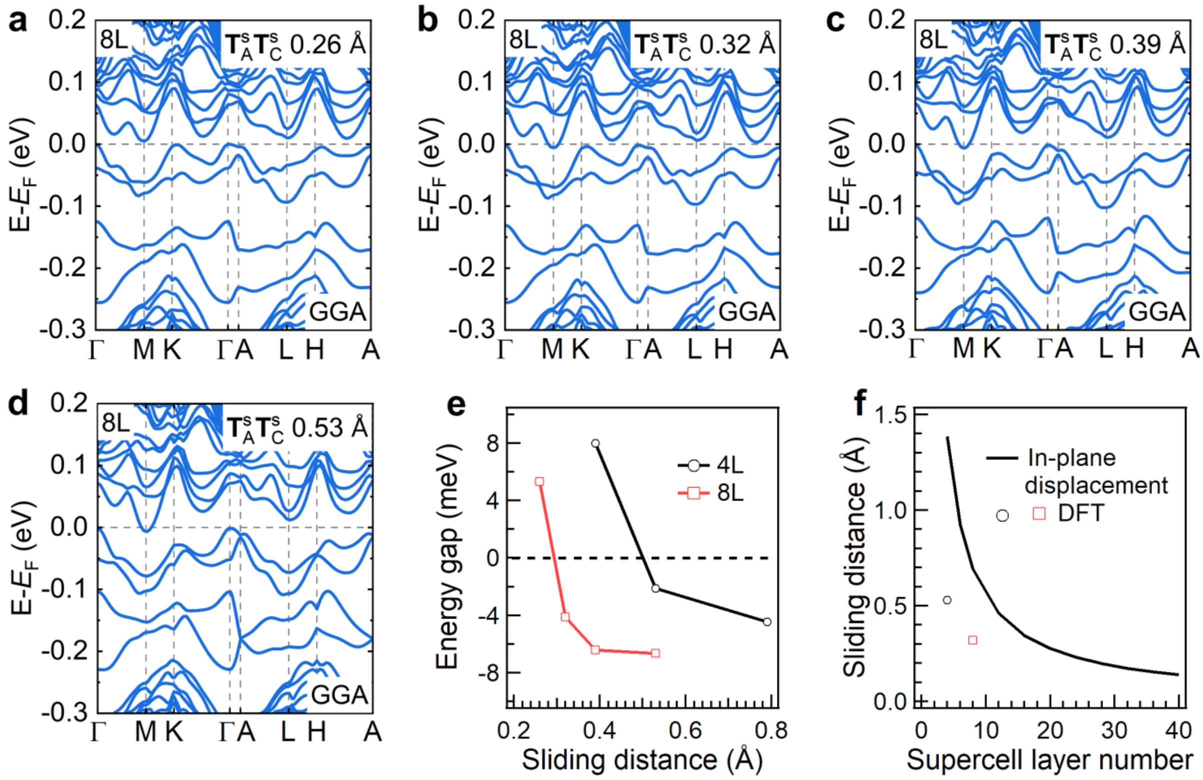

Supplementary Fig. 11. DFT calculated band structures of 8-layer supercell structures. Band structures for $T^S_A T^S_C$ stacking with different sliding distances δ**a** of **a** 0.26Å (1/12**a**), **b** 0.32Å (1/10**a**), **c** 0.39Å (1/8**a**), and **d** 0.53Å (1/6**a**), respectively. **e** The evolution of calculated energy gap as a function of sliding distance δ**a** for the 4- and 8-layer supercell. The gap value in panel **e** is taken from the difference of the VBM and CBM from panel **a-d** and Supplementary Fig. 10**a**-10**c**. The negative gap value indicates a metal state. **f** The evolution of in-plane displacement √3/*n***a** for *n*-layer supercell structures, along with minimum sliding distance value that close the gap.

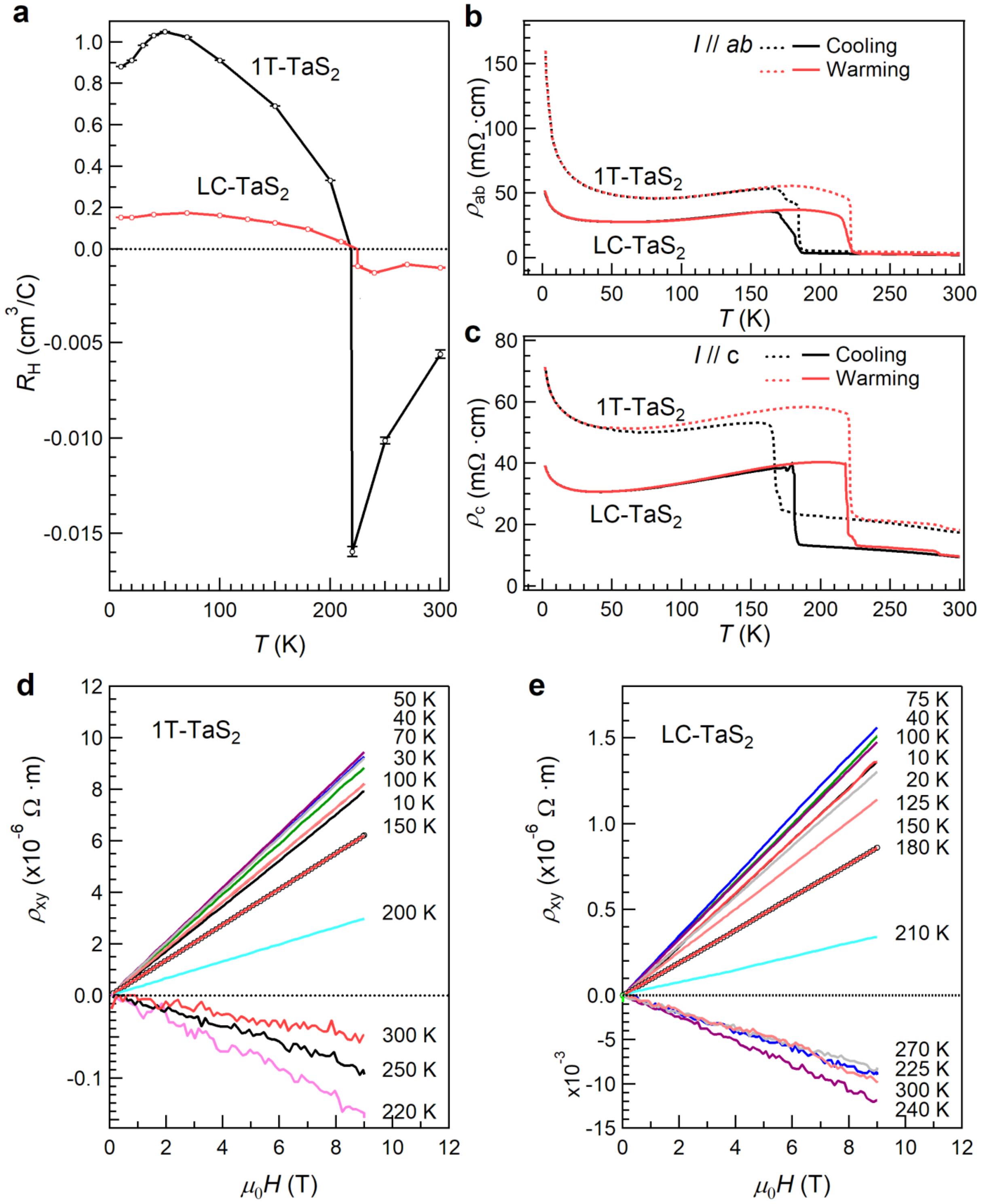

Supplementary Fig. 12. Anisotropic electrical transport properties. a The Hall coefficient R_H for the 1T-TaS_2 and LC-TaS_2 extracted from linear fitting of Hall resistivity ρ_{xy} as a function of the applied magnetic field at various temperatures for **d** 1T-TaS_2 and **e** LC-TaS_2. The error bars in panel **a** are determined from the standard deviation. Temperature dependence of **b** original ρ_{ab} and **c** ρ_c, for 1T-TaS_2 and LC-TaS_2 upon cooling and warming.
